# Präoperative Evaluation erwachsener Patientinnen und Patienten vor elektiven, nicht herz-thoraxchirurgischen Eingriffen: Eine gemeinsame Empfehlung der Deutschen Gesellschaft für Anästhesiologie und Intensivmedizin, der Deutschen Gesellschaft für Chirurgie und der Deutschen Gesellschaft für Innere Medizin

**DOI:** 10.1007/s00101-024-01408-2

**Published:** 2024-05-03

**Authors:** Christian Zöllner, Deutsche Gesellschaft für Anästhesiologie und Intensivmedizin, Andreas Böhmer, Götz Geldner, Jörg Karst, Frank Wappler, Bernhard Zwissler, Matthias Pauschinger, Udo Obertacke, Tim Vilz, Deutsche Gesellschaft für Chirurgie, Deutsche Gesellschaft für Innere Medizin

**Affiliations:** 1 Nürnberg, Deutschland; 2 Wiesbaden, Deutschland; 3 Berlin, Deutschland; 4 Universitätsklinikum Hamburg-Eppendorf, Klinik und Poliklinik für Anästhesiologie, Zentrum für Anästhesiologie und Intensivmedizin, Martinistr. 52, 20246 Hamburg, Deutschland

**Keywords:** Präoperative Evaluation, Kardiales Risiko, Perioperatives Risiko, Zerebrales Risiko, Pulmonales Risiko, Dauermedikation, Preoperative evaluation, Perioperative Risk, Cardiac Risk, Pulmonary Risk, Cerebral risk, Continuous medication

## Abstract

Die 70 Empfehlungen fassen den aktuellen Stand der präoperativen Risikoevaluation von erwachsenen Patientinnen und Patienten vor elektiven, nicht herz-thoraxchirurgischen Eingriffen zusammen. Basierend auf den gemeinsamen Publikationen der deutschen wissenschaftlichen Fachgesellschaften für Anästhesiologie und Intensivmedizin (DGAI), Chirurgie (DGCH) und Innere Medizin (DGIM), die erstmals im Jahr 2010 publiziert und 2017 aktualisiert wurden, sowie der 2022 publizierten europäischen Leitlinie zur präoperativen kardialen Risikoevaluation, findet eine umfassende Neubewertung der Empfehlungen unter Berücksichtigung neuer Erkenntnisse, der aktuellen Literatur sowie aktueller Leitlinien internationaler Fachgesellschaften statt. Die überarbeiteten, fachübergreifenden Empfehlungen sollen ein strukturiertes und gemeinsames Vorgehen in der präoperativen Evaluation der Patientinnen und Patienten ermöglichen. Ziel ist es, eine für die Patientin und den Patienten individualisierte Vorbereitung vor dem operativen Eingriff sicherzustellen und hierdurch die Patientinnen- und Patientensicherheit zu erhöhen. Unter Berücksichtigung eingriffs- und patientinnen- und patientenspezifischer Faktoren, die in der präoperativen Risikoevaluation unabdingbar sind, soll das perioperative Risiko für die Patientin und den Patienten minimiert und die Sicherheit erhöht werden. Die Handlungsempfehlungen sind unter „Allgemeine Prinzipien (A)“, „Erweiterte Diagnostik (B)“ und dem „Präoperativen Umgang mit der Dauermedikation (C)“ zusammengefasst. Erstmals wurde in den vorliegenden Empfehlungen eine Wertung der Einzelmaßnahmen hinsichtlich ihrer klinischen Relevanz gegeben. Durch eine gemeinsame und transparente Absprache sollen eine hohe Patientinnen- und Patientenorientierung unter Vermeidung unnötiger Voruntersuchungen gewährleistet, präoperative Untersuchungsabläufe verkürzt sowie letztlich Kosten eingespart werden. Die gemeinsamen Empfehlungen von DGAI, DGCH und DGIM spiegeln den gegenwärtigen Kenntnisstand sowie die Meinung von Experten wider. Die Empfehlung ersetzt nicht die individualisierte Entscheidung zwischen Patientin und Patient und Ärztin und Arzt über die beste präoperative Strategie und Behandlung.

## Abstract (en)

The 70 recommendations summarize the current status of preoperative risk evaluation of adult patients prior to elective non-cardiothoracic surgery. Based on the joint publications of the German scientific societies for anesthesiology and intensive care medicine (DGAI), surgery (DGCH), and internal medicine (DGIM), which were first published in 2010 and updated in 2017, as well as the European guideline on preoperative cardiac risk evaluation published in 2022, a comprehensive re-evaluation of the recommendation takes place, taking into account new findings, the current literature, and current guidelines of international professional societies. The revised multidisciplinary recommendation is intended to facilitate a structured and common approach to the preoperative evaluation of patients. The aim is to ensure individualized preparation for the patient prior to surgery and thus to increase patient safety. Taking into account intervention- and patient-specific factors, which are indispensable in the preoperative risk evaluation, the perioperative risk for the patient should be minimized and safety increased. The recommendations for action are summarized under “General Principles (A),” “Advanced Diagnostics (B),” and the “Preoperative Management of Continuous Medication (C).” For the first time, a rating of the individual measures with regard to their clinical relevance has been given in the present recommendation. A joint and transparent agreement is intended to ensure a high level of patient orientation while avoiding unnecessary preliminary examinations, to shorten preoperative examination procedures, and ultimately to save costs. The joint recommendation of DGAI, DGCH and DGIM reflects the current state of knowledge as well as the opinion of experts. The recommendation does not replace the individualized decision between patient and physician about the best preoperative strategy and treatment.

## Infobox Interdisziplinäre Empfehlung

*Unter maßgeblicher Mitarbeit von*

- Deutsche Gesellschaft für Anästhesiologie und Intensivmedizin (DGAI)
- Deutsche Gesellschaft für Chirurgie (DGCH)
- Deutsche Gesellschaft für Innere Medizin (DGIM)

## Präambel

Die vorliegenden Empfehlungen richten sich an alle Berufsgruppen, die in der präoperativen Betreuung von Patientinnen und Patienten, die sich einem elektiven, nicht herz-thoraxchirurgischen Eingriff unterziehen müssen, beteiligt sind. Ziel ist es, die standardisierte und Evidenz-basierte präoperative Evaluation dieser Patientinnen und Patienten zu vereinfachen und die perioperative Morbidität und Letalität zu senken. Die Empfehlungen beinhalten die allgemeinen Prinzipien der präoperativen Evaluation (Teil A), die erweiterte Diagnostik (Teil B) sowie den präoperativen Umgang mit Dauermedikationen (Teil C). Es handelt sich um eine Überarbeitung und Aktualisierung der erstmals im Jahre 2010 publizierten und 2017 überarbeiteten Empfehlungen der wissenschaftlichen Deutschen Fachgesellschaften der Anästhesiologie und Intensivmedizin (DGAI), Chirurgie (DGCH) und Innere Medizin (DGIM) ([1, 2]; Infobox 1), die seither im deutschsprachigen Raum eine gute Durchdringung und Akzeptanz erfahren haben [3, 4]. Gleichzeitig finden die aktualisierten Leitlinien der Europäischen Kardiologischen Gesellschaft (ESC) zur präoperativen kardiovaskulären Evaluation von Patientinnen und Patienten vor nicht-kardiovaskulären operativen Eingriffen sowie die ESAIC Empfehlungen zum Einsatz kardialer Biomarker in der präoperativen Risikoevaluation Berücksichtigung [5–7].

### Konsensuseinstufung

Die Empfehlungen wurden als Expertenkonsens der Expertengruppe beschlossen und sollen die Entscheidungen für ein zielgerichtetes präoperatives Management im klinischen Alltag erleichtern. Auch wenn im Einzelfall keine zweifelsfreie, belastbare Evidenz aus prospektiven klinischen Studien vorliegt, wird basierend auf den verfügbaren Daten zwischen drei Empfehlungsgraden unterschieden, deren unterschiedliche Stärke durch die Formulierungen „soll“, „sollte“ und „kann“ ausgedrückt wird (Tab. 1).

**Table 1:** 

Beschreibung				Empfehlungsstärke	Empfehlungsgrad
Empfehlung [Farbcodierung]	Symbol [Farbcodierung]	Empfehlung gegen eine Maßnahme [Farbcodierung]	Symbol [Farbcodierung]		
„Soll“ [dunkles Grün]	⇑⇑ [dunkles Grün]	„Soll nicht“ [dunkles Rot]	⇓⇓ [dunkles Rot]	Starke Empfehlung	A
„Sollte“ [helles Grün]	⇑ [helles Grün]	„Sollte nicht“ [Orange]	⇓ [Orange]	Empfehlung	B
„Kann“/„ist unklar“ [Gelb]	⇔ [Gelb]	„Kann verzichtet werden“/„ist unklar“ [Gelb]	⇔ [Gelb]	Empfehlung offen	0

**Figure 1:**
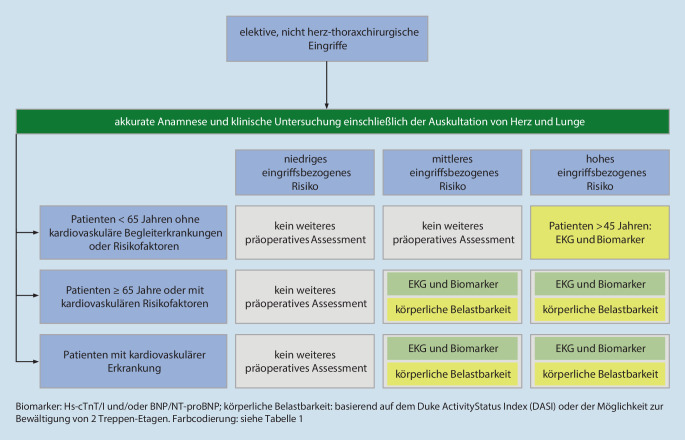

**Figure 2:**
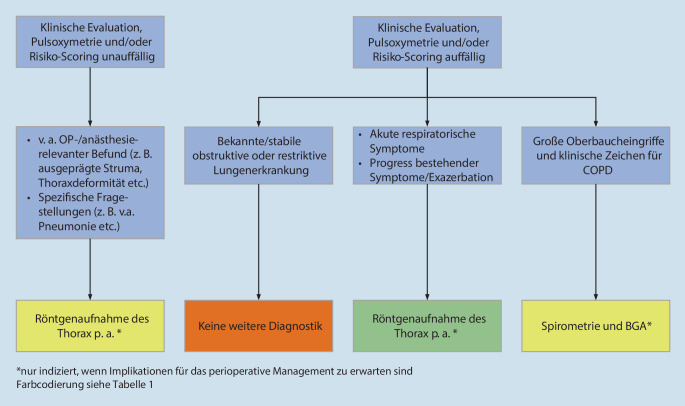

**Figure 3:**
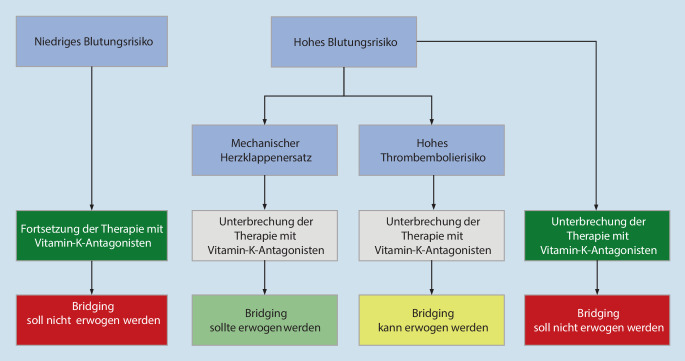

**Figure 4:**
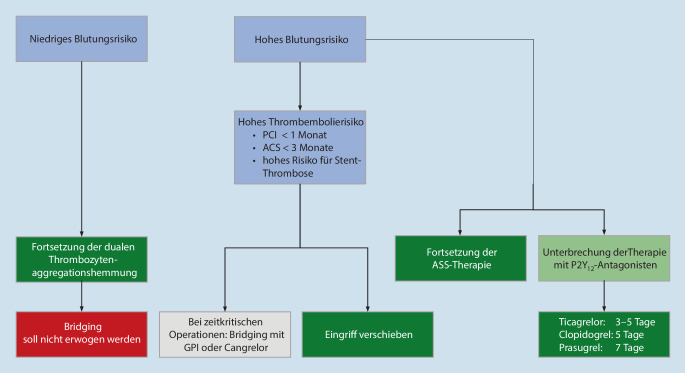

Die angegebene Farbcodierung gilt für die Abb. 1–4.

## A. Allgemeine Prinzipien

Die Zahl der vollstationären Operationen in Krankenhäusern in Deutschland ist im Zeitraum von 2005 bis 2021 von 12,1 Mio. auf 15,8 Mio. gestiegen [8]. Hierbei entfallen 85 % der großen Operationen auf elektive, nicht herz-thoraxchirurgische Eingriffe.

Die präoperative Risikoevaluation umfasst das durch die Art des operativen Eingriffs bedingte Risiko sowie das patientinnen- und patientenbezogene Risiko. Das durch den chirurgischen Eingriff induzierte Risiko ist abhängig von Art, Dauer und Dringlichkeit der Operation. Das patientinnen- und patientenbezogene Risiko einer Operation ist abhängig vom Alter und relevanten Komorbiditäten des Patienten. Zur korrekten Einschätzung des perioperativen Risikos der Patientin bzw. des Patienten soll eine präoperative Evaluation rechtzeitig und in ausreichendem Abstand zum operativen Eingriff erfolgen, so dass die Patientin oder der Patient in Ruhe und reflektiert über seine Einwilligung entscheiden kann [3, 4]. Im BGH-Urteil vom 20. Dezember 2022 (BGB § 630e Abs. 2 S. 1 Nr. 2) wird hierzu festgehalten, dass „der Patient vor dem beabsichtigten Eingriff so rechtzeitig aufgeklärt werden muss, dass er durch hinreichende Abwägung der für und gegen den Eingriff sprechenden Gründe seine Entscheidungsfreiheit und damit sein Selbstbestimmungsrecht in angemessener Weise wahrnehmen kann. Die Bestimmung sieht keine vor der Einwilligung einzuhaltende ‚Sperrfrist‘ vor, deren Nichteinhaltung zur Unwirksamkeit der Einwilligung führen würde; sie enthält kein Erfordernis, wonach zwischen Aufklärung und Einwilligung ein bestimmter Zeitraum liegen müsste. Zu welchem konkreten Zeitpunkt ein Patient nach ordnungsgemäßer – insbesondere rechtzeitiger – Aufklärung seine Entscheidung über die Erteilung oder Versagung seiner Einwilligung trifft, ist seine Sache. Sieht er sich bereits nach dem Aufklärungsgespräch zu einer wohlüberlegten Entscheidung in der Lage, ist es sein gutes Recht, die Einwilligung sofort zu erteilen. Wünscht er dagegen noch eine Bedenkzeit, so kann von ihm grundsätzlich erwartet werden, dass er dies gegenüber dem Arzt zum Ausdruck bringt und von der Erteilung einer – etwa im Anschluss an das Gespräch erbetenen – Einwilligung zunächst absieht“.

Nur durch die Einzelfallentscheidung kann das Selbstbestimmungsrecht der Patientin oder des Patienten in angemessener Weise gewahrt werden (Bürgerliches Gesetzbuch (BGB) § 630e Abs. 2 S. 1 Nr. 2). Gleichzeitig soll es bei ausreichendem Abstand auch möglich sein, die Patientin oder den Patienten bestmöglich auf den elektiven Eingriff vorzubereiten. Die jeweils geltende Rechtsprechung ist zu beachten.

Bei verschobenen elektiven Eingriffen und diagnostischen interventionellen Verfahren muss die Wiederholung der Aufklärung und der Einwilligungserklärung berücksichtigt werden. Grundsätzlich gilt: Je länger die ursprüngliche Aufklärung zurückliegt, desto umfangreicher muss auch die erneute Aufklärung der Patientin oder des Patienten erfolgen. Durch ein Urteil des Bundesgerichtshofs (BGH) kann von einer Wirksamkeit der Einwilligung bis zu 6 Wochen zwischen Aufklärung und Eingriff ausgegangen werden (BGH-Urteil vom 28. Januar 2014, Az: VI ZR 143/13). Der BGH trifft in vorgenannter Entscheidung allerdings keine Beschränkung auf ausschließlich 6 Wochen. Auch wenn die Rechtsprechung bislang keine konkreten Verfallsfristen vorgibt, sollte eine Aufklärung, die länger als drei Monate zurückliegt, kritisch bewertet werden [9]. Wurde die Patientin oder der Patient jedoch mehr als sechs Monate vor dem Eingriff aufgeklärt, muss eine vollständig neue Aufklärung erfolgen (OLG Dresden, Beschluss vom 15. November 2016 – 4 U 57/16). Grundsätzlich gilt, dass eine wirksame Einwilligung erneut eingeholt werden muss, wenn sich im Zeitraum zwischen der erstmaligen Aufklärung und nun geplantem OP-Tag in der Zwischenzeit neue Erkenntnisse ergeben haben, die zu einer veränderten Risiko‑/Nutzenbeurteilung führen. Falls die Patientin oder der Patient weitere Fragen oder keine ausreichende Erinnerung mehr an die Aufklärung zu dem geplanten Eingriff hat, sollte ebenfalls eine erneute Einwilligung eingeholt werden. Diese Umstände zu prüfen ist hierbei Aufgabe des Arztes.

### A.1 Präoperative Evaluation

#### A.1.1 Allgemeines patientinnen- und patientenbezogenes Risiko

Das patientinnen- und patientenbezogene Risiko wird durch das Alter, kardiovaskuläre Risikofaktoren oder akute Erkrankungen und/oder Komorbiditäten bestimmt. Die hierbei erhobenen Befunde sind für die Wahl des Anästhesieverfahrens und die anästhesiologische Behandlung der Patientinnen und Patienten von herausragender Bedeutung.

##### A.1.1.1 Anamnese und körperliche Untersuchung.

Voraussetzung zur Beurteilung des Risikos sind die präoperativ akkurat durchgeführte Anamnese und körperliche Untersuchung (Empfehlung 1) ^**E1**^. Dies schließt eine Blutungsanamnese (vgl. A.2.1) sowie die Selbsteinschätzung der körperlichen Belastbarkeit durch die Patientin bzw. den Patienten ein.

Die präoperative Evaluation sollte zum gleichen Zeitpunkt mit der chirurgischen Aufklärung erfolgen ^**E2**^. Hierdurch können Patientinnen und Patienten mit einer für die Operation oder Anästhesie relevanten Erkrankung oder Vormedikation (z. B. kardiovaskuläre oder pulmonale Medikation, Antikoagulation, Analgetika wie insbesondere Opioide) bereits präoperativ erkannt und für den operativen oder diagnostischen Eingriff optimal vorbereitet werden.

Zu einer adäquaten anästhesiologischen körperlichen Untersuchung gehört insbesondere auch die Untersuchung des Atemwegs zur Erfassung etwaiger Erschwernisse bei der anästhesiologischen Therapie/Behandlung sowie die Inspektion und ggf. Palpation von potenziellen Punktionsstellen für invasive Katheter bzw. für regionalanästhesiologische Verfahren. Anamnese und Untersuchung sollten nach einem standardisierten Schema erfolgen. Weiterführende Untersuchungen sind nicht erforderlich, falls sich keine relevanten, das perioperative Vorgehen beeinflussende Vorerkrankungen erkannt werden.

##### A.1.1.2 Patientinnen und Patienten ohne kardiovaskuläre Begleiterkrankungen oder Risikofaktoren.

Patientinnen und Patienten die keine Zeichen, Symptome oder Anamnese für kardiovaskuläre Erkrankungen oder Risiken haben, können bei niedrigem oder mittlerem eingriffsbezogenem Risiko ohne weitere präoperative Risikoeinschätzung anästhesiologisch behandelt werden. Vor Operationen mit hohem eingriffsbezogenen Risiko kann bei Patientinnen und Patienten über 45 Jahre die Anfertigung eines EKG und die Bestimmung von kardialen Biomarkern erwogen werden (Abb. 1). Patientinnen und Patienten ohne Zeichen oder Symptome einer kardiovaskulären Risikoerkrankung, aber mit einer familiären Disposition für das Auftreten von Kardiomyopathien sollen bereits präoperativ ein EKG und eine echokardiographische Diagnostik erhalten ([5, 6, 10]; Abb. 1).

##### A.1.1.3 Patientinnen und Patienten mit kardiovaskulären Risikofaktoren.

Patientinnen und Patienten mit kardiovaskulären Risikofaktoren (z. B. Dyslipidämie, arterielle Hypertonie, Diabetes mellitus, Adipositas, Rauchen) haben ein erhöhtes Risiko für eine nicht diagnostizierte kardiovaskuläre Erkrankung und perioperative Komplikationen [11, 12]. Bei Operationen mit mittlerem und hohem eingriffsbezogenen Risiko sollte eine erweiterte präoperative Risikoevaluation (EKG, Kardiale Biomarker) durchgeführt werden [5, 6]. Die körperliche Belastbarkeit kann, basierend auf der Möglichkeit zur Bewältigung von 2 Treppen-Etagen, erhoben werden (Abb. 1).

##### A.1.1.4 Patientinnen und Patienten mit manifesten kardiovaskulären Erkrankungen.

Operationen bei Patientinnen und Patienten mit manifesten kardiovaskulären Erkrankungen können die perioperative Mortalität erhöhen. Diese Eingriffe sollten grundsätzlich erst nach einer differenzierten kardiovaskulären Risikoabklärung unter Berücksichtigung des kardiovaskulären Risikos, Art des chirurgischen Eingriffs und der Dringlichkeit der elektiven Operation erfolgen. Die Evaluation kann eine invasive oder nicht-invasive Diagnostik sowie therapeutische Interventionen vor dem elektiven Eingriff beinhalten. Bei einem akuten Koronarsyndrom sollen die aktuellen Leitlinien zur Behandlung von Patientinnen und Patienten im nicht-operativen Bereich zur Anwendung kommen. Bei Operationen mit mittlerem und hohem eingriffsbezogenen Risiko sollte eine erweiterte präoperative Risikoevaluation (EKG, Kardiale Biomarker) durchgeführt werden ([5, 6, 13, 14]; Abb. 1). Das geeignete Vorgehen erfordert eine interdisziplinäre Absprache.

##### A.1.1.5 Frailty.

Die Altersgebrechlichkeit (Frailty) ist die Entität, die am stärksten mit postoperativen Komplikationen, Morbidität und Letalität assoziiert ist [15]. Bei betroffenen Patientinnen und Patienten ist wegen des hohen Risikos ein adjustiertes perioperatives Management indiziert. Die präoperative Evaluation älterer Patientinnen und Patienten (> 70 Jahre), die sich einer Operation mit mittlerem oder hohem eingriffsbezogenen Risiko unterziehen, kann durch ein Frailty Screening ergänzt werden ^**E3**^ [5, 6].

Frailty kumuliert mehrere Alters-assoziierte perioperative Risikofaktoren zu einem Gesamtrisiko, das Aufschluss über den funktionellen Status und die Vulnerabilität älterer Menschen gibt und dem „biologischen“ Alter eines Individuums am ehesten entspricht. Hierzu können z. B. die Clinical Frailty Scale oder der Risk Analysis Index (RAI-C) erhoben werden [16].

Das erhöhte perioperative Risiko sollte im Rahmen von partizipativer Entscheidungsfindung bereits bei der Indikationsstellung zur Operation berücksichtigt werden ^**E4**^. Ein perioperatives Behandlungskonzept für Patientinnen und Patienten mit Frailty sollte besonderen Wert auf prähabilitative Elemente (z. B. präoperative Ernährungssubstitution bei Mangelernährung, präoperatives Atemtraining) und Delirprävention legen (u. a. Aufklärung von Patientinnen und Patienten, Einbindung von Angehörigen, Fast-track-Konzepte, Neuromonitoring zur Vermeidung zu tiefer Narkose). Der perioperative Umgang mit dieser Patientinnen- und Patientengruppe sollte darauf abzielen, Komplikationen vorzubeugen (u. a. Vermeidung von potenziell inadäquater Medikation, Vermeidung unnötiger Nüchternheit/Dehydratation, Frühmobilisation) [17]. Eine Sensibilisierung des medizinischen Personals für die Bedeutung und den Umgang mit Frailty ist hierfür Voraussetzung ^**E5**^ (Tab. 2).

**Table 2:** 

	Empfehlung	Empfehlungsgrad	
E1	Alle PatientInnen zur präoperativen Evaluation vor elektiven Eingriffen SOLLEN eine akkurate Anamnese und körperliche Untersuchung einschließlich der Auskultation von Herz und Lunge erhalten.	A	⇑⇑
E2	Die präoperative Evaluation SOLLTE zum gleichen Zeitpunkt mit der chirurgischen Aufklärung erfolgen [5, 6].	B	⇑
E3	Bei PatientInnen über 70 Jahren KANN Frailty präoperativ bei Eingriffen mit mittlerem und hohem eingriffsbezogenem Risiko erfasst werden [5, 6].	0	⇔
E4	PatientInnen mit Frailty benötigen ein adjustiertes perioperatives Management, in dem partizipative Entscheidungsfindung, Prähabilitation und Delirprävention eine vorrangige Rolle spielen SOLLTEN.	B	⇑
E5	Medizinisches Personal SOLLTE hinsichtlich der Bedeutung von Frailty und über entsprechende Behandlungskonzepte geschult werden.	B	⇑

#### A.1.2 Allgemeines eingriffsbezogenes Risiko

Das eingriffsbezogene Risiko berücksichtigt die Art, Dauer und Dringlichkeit des chirurgischen Eingriffs und bezieht sich in den meisten Studien auf ein 30 Tage Risiko für Tod durch kardiovaskuläre Erkrankungen, Myokardinfarkt oder Schlaganfall [18]. Basierend auf der Art des Eingriffs oder der Intervention wird das Risiko mit einem niedrigen (< 1 %), mittleren (1–5 %) oder hohen eingriffsbezogenen Risiko (> 5 %) klassifiziert (siehe Tab. 3). Neben dem eingriffsbezogenem Risiko können durch Kombination mit einfach zu erhebenden Parametern-, wie ASA-Klassifikation, Dringlichkeit des Eingriffs, chirurgisches Risiko [19] oder mit Parametern wie Alter, kardiovaskuläre Begleiterkrankungen, Art und Dringlichkeit des Eingriffs, Schlaganfall in der Anamnese sowie Serum-Natrium, Kreatinin und Hamatokrit [20] Risiko-Indizes ermittelt werden, die bereits präoperativ eine hohe Vorhersagekraft für die 30-Tage Letalität liefern. Diese bereits präoperativ durchgeführte Erhebung kann wichtige Informationen bei der Planung operativer Eingriffe, Entwicklung risikoreduzierender Strategien und zur Verbesserung der Behandlungsqualität liefern.

**Table 3:** 

Niedriges eingriffsbezogenes Risiko (< 1 %)	Mittleres eingriffsbezogenes Risiko (1–5 %)	Hohes eingriffsbezogenes Risiko (> 5 %)
Chirurgie oberflächlicher Strukturen	Chirurgie intraperitonealer Organe	Chirurgie der Aorta und großer Gefäße
Chirurgie der Zähne	Karotis-Endarterektomie – symptomatisch und asymptomatisch	Offene peripherarterielle Gefäßeingriffe und Amputationen an der unteren Extremität
Chirurgie der Schilddrüse	Karotis-Stenting – asymptomatisch	Karotis-Stenting – symptomatisch
Chirurgie des Auges	Endovaskuläre Aortenchirurgie	Gefäß-Revaskularisationen oder Amputationen der Extremitäten
Plastisch-rekonstruktive Chirurgie	Chirurgie intraperitonealer Organe	Chirurgie des Duodenums oder Pankreas
Chirurgie der Mamma	Große orthopädische- und Wirbelsäulenchirurgie	Chirurgie bei Darmperforationen
Kleine gynäkologische Chirurgie	Große urologische und gynäkologische Chirurgie	Chirurgie der Nebenniere
Kleine gynäkologische Chirurgie	Nierentransplantation	Chirurgie der Leber oder Gallengänge
Kleine gynäkologische Chirurgie	Kleine intrathorakale Chirurgie	Ösophagektomie
	Peripher arterielle Angioplastie	Chirurgie der Nebenniere
		Zystektomie (total)
		Pneumonektomie (VATS oder offene Chirurgie)
		Leber-/Lungentransplantation
		Totale Zystektomie

*VATS* video-assisted thoracoscopic surgery

* Kardiovaskuläres Risiko für Tod, Myokardinfarkt oder Schlaganfall innerhalb von 30 Tagen

#### A.1.3 Multimodales Perioperatives Management (mPOM)

##### A.1.3.1 Prähabilitation.

Unter Prähabilitationsmaßnahmen werden verschiedene Interventionen (Tab. 4) zusammengefasst, die den Zustand der Patientin bzw. des Patienten vor einer Operation optimieren und bereits präoperativ in Erwägung gezogen werden können ^**E6**^ [21]. Interventionen wie ausgewogene Ernährung, Alkohol- und Nikotinkarenz sowie körperliche Aktivität sollten als Standardmaßnahmen vor jeder Operation durchgeführt werden ^**E7**^.

**Table 4:** 

Prähabilitation	Präoperativ
PatientInneninformation/-edukation	Thromboseprophylaxe
Unterstützung bei Alkohol- und Nikotinkarenz	Kurze Nüchternheitsphase und Zugang zu kohlenhydratreichen Drinks
Ernährungsberatung mit Therapie einer Malnutrition, ggfs. Nahrungsergänzung	Darmvorbereitung in Abhängigkeit vom geplanten Eingriff
Körperliches Training	Sedierende Prämedikation zur Anxiolyse im Einzelfall in Betracht ziehen
Psychosomatische/psychoonkologische Mitbetreuung	Antibiotikaprophylaxe vor Schnitt
Anämiebehandlung	Validierte Risikostratifizierung
Kognitives Training	Anpassung von Analgetika

##### A.1.3.2 Fast Track Chirurgie.

Das Prinzip der „Fast Track (FT) Chirurgie“ wurde 1995 erstmalig bei kolorektalen Resektionen beschrieben und umfasst heute im Rahmen eines „Multimodalen Perioperativen Managements (mPOM)“ eine Vielzahl verschiedener Einzelmaßnahmen, die die postoperative Morbidität und die Krankenhausverweildauer signifikant senken können [22]. Auch die Behandlungskosten konnten gesenkt werden [23–26]. Ein Multimodales Perioperatives Behandlungskonzept soll bereits präoperativ begonnen werden und erfordert die strenge Adhärenz zu den einzelnen Maßnahmen ^**E8**^ [27]. Hierzu zählen in der unmittelbar präoperativen Phase kurze präoperative Nüchternheitsphasen, die Antibiotikaprophylaxe, die Beschränkung der präoperativen Nahrungs- bzw. Flüssigkeitskarenz auf maximal 6 h bzw. 2 h, der Verzicht auf Sonden und Katheter sowie in der Viszeralchirurgie die Darmvorbereitung. Eine detaillierte Darstellung zu diesem Thema ist in der S3-Leitlinie „Perioperatives Management bei gastrointestinalen Tumoren (POMGAT)“ zu finden ([28]; Tab. 5).

**Table 5:** 

	Empfehlung	Empfehlungsgrad	
E6	Intensivierte Prähabilitationsmaßnahmen KÖNNEN präoperativ in Erwägung gezogen werden.	0	⇔
E7	Präoperativ SOLLTEN Nikotin- und Alkoholkarenz, ausgewogene Ernährung sowie körperliche Aktivität empfohlen werden.	B	⇑
E8	Ein Fast Track- oder Multimodales Perioperatives Behandlungskonzept SOLL präoperativ begonnen werden.	A	⇑⇑

#### A.1.4 Patientinnen- und Patienteninformation und Aufklärung

##### A.1.4.1 Patientinnen- und Patienteninformation.

Die koordinierende Rolle der Anästhesiologin und des Anästhesiologen zwischen den Fachdisziplinen berücksichtigt die Bedürfnisse der Patientin bzw. des Patienten. Die Patientin bzw. den Patienten betreffende Behandlungsentscheidungen müssen gemeinsam mit dem Behandlungsteam und der Patientin bzw. dem Patienten getroffen werden. Dieses Vorgehen führt zu einer höheren Zufriedenheit der Patientin bzw. des Patienten und verhindert präoperative Konflikte und Ängste. Die Information sollte hierbei nicht nur in mündlicher, sondern auch in schriftlicher Form oder unter Hinzuziehung multimedialer Systeme (DVD, interaktive Videoclips etc.) erfolgen.

Die Prävalenz präoperativer Ängste vor chirurgischen Eingriffen variiert in Untersuchungen von 27–80 % [29] und beeinflusst direkt das postoperative Outcome der Patientinnen und Patienten [30]. Die hierfür verantwortlichen Faktoren sind multidimensional und müssen individualisiert erhoben werden.

Zur weiteren Planung des perioperativen Behandlungsablaufes muss die Patientin bzw. der Patient in die Entscheidung über das Narkoseverfahren und die perioperative Schmerztherapie mit einbezogen werden [31]. Änderungen in der präoperativen Medikation sollen der Patientin bzw. dem Patienten in verbaler und schriftlicher Weise zur Verfügung gestellt werden ^**E9**^.

##### A.1.4.2 Fernaufklärung.

In der Gesetzesbegründung zu § 630 e BGB, sog. Patientenrechtegesetz (BT-Drucks. 17/10488 vom 15.08.2012) ist hinterlegt, dass die Patientenaufklärung in einem persönlichen Gespräch mit dem Behandelnden und der Möglichkeit zu Rückfragen erfolgen muss. Die Rechtsprechung des BGH sieht vor, dass „die Aufklärung in einfach gelagerten Fällen auch fernmündlich erfolgen kann“ (BGH 15.06.2010, Az. VI ZR 204, 2009). Gerade im Rahmen der Covid19-Pandemie wurden neue Verfahren der Patientinnen- und Patientenaufklärung entwickelt. Die digitalisierte, rechtskonforme und videoassistierte Fernaufklärung (DRVF) hat sich in den zurückliegenden Jahren als neues Verfahren der Patientinnen- und Patientenaufklärung etabliert und weiterentwickelt. Die DRVF führt insbesondere bei einer Verlagerung operativer Gesundheitsleistungen aus dem stationären in den ambulanten Sektor zu einer Steigerung der Patientinnen- und Patientenzufriedenheit und reduziert den Aufwand für die Patientin bzw. den Patienten. Sie ermöglicht der aufklärenden Ärztin bzw. dem aufklärenden Arzt, sich einen Gesamteindruck der Patientin bzw. des Patienten zu verschaffen und gewisse Evaluationen (wie z. B. den schwierigen Atemweg) fernmündlich durchzuführen. Hierfür kommen ausschließlich Patientinnen und Patienten in Frage, die sich einem niedrig-komplexen Routineeingriff unterziehen, die erforderlichen technischen Möglichkeiten besitzen sowie kognitiv in der Lage und mit dem Verfahren einverstanden sind ^**E10**^. Die novellierte Musterberufsordnung bietet die Möglichkeit einer ausschließlichen Fernbehandlung [32, 33]. In der Gesetzesbegründung zum Digitale-Versorgungs-Gesetz (DVG; BT-Drucks. 19/13438 vom 23.09.2019) wird festgelegt, dass „das traditionell übliche persönliche Gespräch in der Praxis des Behandelnden heute durch die Verwendung von Telekommunikationsmitteln ersetzt werden kann, ohne dass Patient und Behandelnder sich in den gleichen Räumlichkeiten aufhalten müssen. Gleiches gilt für die Aufklärungspflicht des Behandelnden gegenüber dem Patienten hinsichtlich Art, Umfang, Durchführung, zu erwartende Folgen und Risiken einer einwilligungsbedürftigen medizinischen Maßnahme“. Die Einführung eines detaillierten Konzeptes in Absprache mit den Rechtsabteilungen des Krankenhausträgers wird hierbei empfohlen (Tab. 6).

**Table 6:** 

	Empfehlung	Empfehlungsgrad	
E9	Individualisierte Anweisungen zur medikamentösen Therapie für PatientInnen SOLLEN präoperativ in verbaler und schriftlicher Form erfolgen [5, 6, 10].	A	⇑⇑
E10	Bei einfachen chirurgischen Eingriffen KANN in Erwägung gezogen werden, die präoperative Aufklärung als Fernaufklärung durchzuführen.	0	⇔

### A.2 Einschätzung des perioperativen Risikos

Perioperative Komplikationen können vor allem das Herz-Kreislauf-, das respiratorische und das zerebrale System betreffen. Dieses individuelle Risiko soll bereits präoperativ abgeschätzt werden.

#### A.2.1 Perioperatives kardiovaskuläres Risiko

In der Abschätzung des perioperativen kardialen Risikos für die Entwicklung eines schwerwiegenden kardiovaskulären Ereignisses („major adverse cardiac event; MACE“) sind im Wesentlichen 4 Faktoren ausschlaggebend:

- Vorliegen einer akuten symptomatischen Herzerkrankung
- das kardiovaskuläre Risikoprofil der Patientin bzw. des Patienten
- die kardiale Belastbarkeit der Patientin bzw. des Patienten
- die kardiale Risikoeinstufung des geplanten operativen Eingriffes

Akut symptomatische kardiale sowie auch extrakardiale Erkrankungen bzw. Befunde, die das Risiko für die Entwicklung schwerwiegender perioperativer kardiovaskulärer Ereignisse erhöhen, sind in Tab. 7 aufgelistet und sollen vor einem elektiven Eingriff ausgeschlossen werden ^**E11**^.

**Table 7:** 

*Reduzierte körperliche Belastbarkeit*	Metabolische Äquivalente (MET) < 4
*Akutes Koronarsyndrom (ACS)* *Chronisches Koronarsyndrom (CCS)*	Myokardinfarkt in der Vorgeschichte Instabile Angina pectoris Schwere Angina pectoris (CCS III–IV)
*Dekompensierte Herzinsuffizienz*	NYHA IV oder aktuelle Symptomverschlechterung oder Erstmanifestation
*Relevante Herzklappenerkrankungen*	Hochgradige Aortenklappenstenose (mittlerer Gradient > 40 mm Hg, AÖF < 1 cm^2^) Hochgradige, symptomatische Mitralklappeninsuffizienz Schwere Mitralklappenstenose (fortschreitende Belastungsdyspnoe bzw. Zeichen der Herzinsuffizienz)
*Signifikante Herzrhythmusstörungen*	AV-Block II° Typ Mobitz, AV-Block III° Symptomatische ventrikuläre Arrhythmie Symptomatische Bradykardie/Tachykardie
*Extrakardiale Risikofaktoren*	Chronische Niereninsuffizienz (Serumkreatinin > 2 mg/dl oder Kreatinin Clearence < 60 ml/min/1,73 m^2^) Vorgeschichte eines zerebralen Ereignisses oder einer transitorischen ischämischen Attacke Insulinpflichtiger Diabetes mellitus Eingeschränkte Lungenfunktion Adipositas (BMI ≥ 30 kg/m^2^) Anämie (Frauen Hb < 12 g/dl; Männer Hb < 13 g/dl) [35]

Verschiedene Scores stehen zur Verfügung, mit denen das Risiko der Entwicklung eines MACE präoperativ ermittelt werden kann. Aufgrund der leichten Anwendbarkeit und der guten Validierung sollte zur Abschätzung des kardialen Risikos bevorzugt der Revised Cardiac Risk Index (RCRI) nach Lee [36] eingesetzt werden (siehe Tab. 8), der auch die Parameter enthält, welche als „Risikofaktoren“ oftmals in Algorithmen Verwendung finden ^**E12**^.

**Table 8:** 

	**Punkte**
Herzinsuffizienz	1
Chronisches Koronar Syndrom (CCS)	1
Zerebrale Insuffizienz (Apoplex oder TIA)	1
Insulinpflichtiger Diabetes mellitus	1
Kompensierte Niereninsuffizienz mit einem Serum Kreatinin > 2 mg/dl	1
**Punktzahl**	**MACE-Risiko (%)**
0	0,4
1	0,9
2	6,6
≥ 3	11

Die körperliche Belastbarkeit ist ein relevanter prognostischer Faktor für das Risiko postoperativer Komplikationen bei großen, elektiven nicht-herzchirurgischen Eingriffen, einschließlich pulmonaler oder kardialer Komplikationen und Tod nach 30 Tagen [37]. Aus diesem Grund sollen die reduzierte körperliche Belastbarkeit und bei über 65-jährigen Patientinnen und Patienten auch eine chronische Niereninsuffizienz oder Anämie vor elektiven Eingriffen ausgeschlossen werden ^**E13**^. Um den Energieverbrauch verschiedener körperlicher Aktivitäten vergleichen zu können, wurde bisher die körperliche Belastbarkeit mit Hilfe von metabolischen Äquivalenten (metabolic equivalent of task, MET) quantifiziert. Auf Grund neuer Daten [38] wird die differenzierte Quantifizierung von metabolischen Äquivalenten durch die subjektive Selbsteinschätzung der Belastbarkeit durch die Patientinnen und Patienten ersetzt. Dies entspricht der anamnestischen Frage nach der Möglichkeit zur Bewältigung von 2 Treppen-Etagen, wobei in vorangegangenen Studien jede Treppe aus 18–21 Stufen unterschiedlicher Höhe bestand ^**E14**^ [37, 39, 40]. Diese körperliche Aktivität entspricht einer metabolischen Rate von > 4 Metabolischen Äquivalenten und ist in Studien mit postoperativen kardialen und pulmonalen Komplikationen sowie der 30 Tage Letalität assoziiert [37]. Diese Selbsteinschätzung der Belastbarkeit ist vor allem bei Eingriffen mit mittlerem und hohem Risiko zu nutzen. Der Test kann aber die funktionellen aeroben und anaeroben Kapazitäten und Bedürfnisse der Patientinnen und Patienten, die für einen operativen Eingriff erforderlich sind, nicht vollumfänglich abbilden ([40]; Tab. 9).

**Table 9:** 

	Empfehlung	Empfehlungsgrad	
E11	Das Vorliegen einer akut-symptomatischen Herzerkrankung SOLL vor einer geplanten Operation ausgeschlossen werden.	A	⇑⇑
E12	Präoperativ SOLLTE bekannt sein, ob und welche Risikofaktoren des Lee-Index vorliegen.	B	⇑
E13	Die reduzierte körperliche Belastbarkeit und bei über 65-jährigen PatientInnen eine chronische Niereninsuffizienz oder Anämie SOLLEN ebenfalls vor einem elektiven Eingriff ausgeschlossen werden.	A	⇑⇑
E14	Insb. vor Operationen mit mittlerem und hohem eingriffsbezogenen Risiko SOLLTE die Selbsteinschätzung der PatientInnen nach der körperlichen Belastbarkeit beim Steigen von Zwei-Etagen-Treppen erfragt werden.	B	⇑

#### A.2.2 Perioperatives pulmonales Risiko

Ziel der präoperativen Evaluation der respiratorischen Funktionen und des pulmonalen Risikos ist die Reduktion postoperativer pulmonaler Komplikationen (PPC).

Hierbei stehen die Anamnese, die körperliche Untersuchung, die pulsoxymetrische Messung der Sauerstoffsättigung sowie die Erhebung einfacher klinischer Risikoscores zur Vorhersage von postoperativen pulmonalen Komplikationen im Vordergrund.

Apparative Verfahren (Thoraxröntgen, Spirometrie, Blutgasanalyse) sind hingegen nur in seltenen Fällen begleitend zu der klinischen Evaluation erforderlich ^**E15**^ [41–43]. Generell sollten apparative diagnostische Verfahren nur angestrebt werden, wenn die Vortestwahrscheinlichkeit für einen auffälligen Befund ausreichend hoch ist und sich aus einem pathologischen Befund direkte Konsequenzen für das perioperative Management der Patientinnen und Patienten ergeben.

Eine Vielzahl klinischer Risikoscores wurde für die präoperative Vorhersage von postoperativen pulmonalen Komplikationen (ARISCAT [44]), postoperativer respiratorischer Insuffizienz (PERISCOPE-PFR [45]), Reintubation (SPORC [46]) oder ARDS (SLIP‑2 Score [47]) entwickelt. In der Praxis hat sich für die präoperative Vorhersage von postoperativen pulmonalen Komplikationen insbesondere der ARISCAT Score ( s. Tab. 10) etabliert [48], auch da er bis dato als einziger Score für die Vorhersage von postoperativ pulmonalen Komplikationen validiert wurde [49]. Der ARISCAT Score soll bei Patientinnen und Patienten mit pulmonalen Erkrankungen bereits präoperativ erhoben werden ^**E16**^ (Tab. 10).

**Table 10:** 

**Faktoren**		**Punkte**
*Alter (Jahre)*		
51–80 Jahre		3
> 80 Jahre		16
*Präoperative SpO*_*2*_* (%)*		
91–95		8
≤ 90		24
*Respiratorischer Infekt im letzten Monat*		17
*Präoperative Anämie (Hb ≤* *10* *g/dl)*		11
*Eingriffsort*		
Oberbauch		15
Intrathorakal		24
*OP-Dauer (Stunden)*		
2–3		16
> 3		23
*Notfalleingriff*		8
**Risiko für PPC während des Krankenhausaufenthaltes**		
Niedrig	Risiko 1,6 %	< 26 Punkte
Mittel	Risiko 13,3 %	26–44 Punkte
Hoch	Risiko 42,1 %	≥ 45 Punkte

*SpO*_*2*_ pulsoxymetrisch gemessene Sauerstoffsättigung, *ARISCAT* Assess Respiratory Risk in Surgical Patients in Catalonia

Patientinnen und Patienten mit einem Obstruktiven Schlafapnoe Syndrom (OSAS) weisen ein erhöhtes Risiko für schwierige Maskenbeatmung und/oder Intubation, postoperativen Atemwegsobstruktionen sowie Komplikationen, die sich auf Komorbiditäten zurückführen lassen, auf. Die Verwendung validierter Fragebögen für ein OSA-Screening sollte zur Einschätzung postoperativer pulmonaler Komplikationen in Betracht gezogen werden [41]. Der STOP-BANG Fragebogen [50] ist hierbei aktuell der sensitivste, spezifischste und am besten validierte Score ^**E17**^ ([41]; Tab. 11).

**Table 11:** 

	Empfehlung	Empfehlungsgrad	
E15	Bei lungengesunden PatientInnen SOLLEN apparative Verfahren zur pulmonalen Diagnostik NICHT zum Einsatz kommen.	A	⇓⇓
E16	ARISCAT eignet sich zur Vorhersage postoperativer pulmonaler Komplikationen und SOLL bei PatientInnen mit pulmonalen Erkrankungen erhoben werden.	A	⇑⇑
E17	Der STOP-BANG Fragebogen sollte zur Identifizierung von OSA Patienten und zur Einschätzung des postoperativen pulmonalen Risikos angewandt werden.	B	⇑

*SpO*_*2*_ pulsoxymetrisch gemessene Sauerstoffsättigung, *ARISCAT* Assess Respiratory Risk in Surgical Patients in Catalonia, *OSAS* Obstruktives Schlafapnoe Syndrom

#### A.2.3 Perioperatives zerebrales Risiko

Perioperative Komplikationen wie Schlaganfall, Delir und neurokognitive Störungen sind häufig und für eine erhöhte Morbidität und Sterblichkeit verantwortlich [51, 52]. Der perioperative Schlaganfall ereignet sich bei 0,1–1 % aller operierten Patientinnen und Patienten, wobei hier neben herz-thoraxchirurgischen auch intrakraniell neurochirurgische und gefäßchirurgische Eingriffe oberhalb des Aortenbogens ausgenommen werden müssen, und geht mit einem 8‑fach erhöhten Sterblichkeitsrisiko, verglichen mit Patientinnen und Patienten ohne Schlaganfall, einher [53]. Das Risiko für einen perioperativen Schlaganfall ist bis zu einem Jahr nach dem Eingriff erhöht [54].

Bei älteren Patientinnen und Patienten tritt ein postoperatives Delir bei bis zu 65 % aller Patientinnen und Patienten auf, während die postoperative kognitive Dysfunktion mit einer Inzidenz zwischen 17 und 43 % aller Patientinnen und Patienten, die sich einem nicht herz-thoraxchirurgischen Eingriff unterziehen müssen, angegeben wird [52, 55]. Die bisher publizierten Empfehlungen zur präoperativen Evaluation zeigen eine Übereinstimmung mit einem hohen Grad an Evidenz.

##### A.2.3.1 Perioperativer Schlaganfall.

Das individuelle Risiko für einen Schlaganfall soll bei Patientinnen und Patienten mit Risikofaktoren bereits präoperativ ermittelt werden ^**E18**^. Im Falle eines erhöhten Risikos können präoperativ unterschiedliche Instrumente zur Quantifizierung des Risikos für einen perioperativen Schlaganfall (Stroke after Surgery (STRAS); AHA/ACC ASCVD Risk Calculator, Revised Cardiac Risk Index (RCRI), ATRIA Score) herangezogen werden ^**E19**^ [54]. Patientinnen und Patienten mit einer transitorischen ischämischen Attacke (TIA) oder einem Schlaganfall in den zurückliegenden 6 Monaten sollen präoperativ ein neurologisches Konsil erhalten sowie einer bildgebenden und neurovaskulären Diagnostik unterzogen werden [5, 6].

Die Frage, ab wann elektive Operationen nach einem Schlaganfall frühestens durchgeführt werden sollen, wird in der Literatur kontrovers diskutiert. Die zerebrale Autoregulation erscheint insbesondere in den ersten 3 Monaten nach dem Ereignis eingeschränkt [56]. In Empfehlungen anästhesiologischer [57] und kardiologischer Leitlinien [53] sollen elektive Eingriffe frühestens 9 Monate nach dem Schlaganfallereignis durchgeführt werden ^**E20**^. Bei Eingriffen, die eine neunmonatige Wartezeit nicht zulassen, ist eine individualisierte, interdisziplinäre Risikoabwägung hinsichtlich eines früheren Operationszeitpunktes durchzuführen.

##### A.2.3.2 Perioperative kognitive Störungen: Postoperatives Delir und postoperative kognitive Dysfunktion.

Das individuelle Risiko für eine neurokognitive Störung soll bei Patientinnen und Patienten mit Risikofaktoren (z. B. präoperative Nüchternheit und Dehydratation, Dauermedikation von anticholinergen Substanzen, Gebrechlichkeit, vorbestehende kognitive Einschränkungen und Mangelernährung) bereits präoperativ ermittelt werden ^**E18**^ [51]. Patientinnen und Patienten mit erhöhtem Risiko für perioperative kognitive Störungen können präoperativ einem validierten neurokognitiven Screening unterzogen werden (z. B. Mini-Cog) ([58, 59]; Tab. 12).

**Table 12:** 

	Empfehlung	Empfehlungsgrad	
E18	Das individuelle Risiko für einen perioperativen Schlaganfall oder eine neurokognitive Störung SOLL präoperativ evaluiert werden.	A	⇑⇑
E19	PatientInnen mit einem postoperativ erhöhten Risiko für einen Schlaganfall oder eine neurokognitive Störung KÖNNEN präoperativ einem validierten Screening unterzogen werden.	0	⇔
E20	Der Abstand zwischen einem stattgehabten Schlaganfall und einer elektiven Operation SOLL > 9 Monate betragen.	A	⇑⇑

#### A.2.4 Perioperatives Risiko für starke Schmerzen und schmerzbedingte Funktionseinschränkungen

##### A.2.4.1 Chronischer Schmerz und Opioidvormedikation.

Chronischer Schmerz ist eine häufige Komorbidität (ca. 27 % Betroffener in der Bevölkerung in Deutschland, ca. 50 % der Patienten im Krankenhaus) und konsistenter Risikofaktor für starke postoperative Schmerzen. Damit sind häufig postoperativ funktionelle Einschränkungen, verzögerte Mobilisation, erhöhter Disstress und eine längere Krankenhausverweildauer assoziiert. Die bereits vorbestehende Langzeiteinnahme von Opioiden stellt einen weiteren unabhängigen Risikofaktor für perioperative Komplikationen dar. Deshalb soll zusätzlich zu jeder präoperativen Risikoevaluation auch eine Schmerzanamnese erhoben werden [31, 60].

Im Rahmen der präoperativen Risikoevaluation sind insbesondere Wirkstoff und Dosis einer bereits vorbestehenden Opioidtherapie zu erheben, zu dokumentieren und im perioperativen Management zu berücksichtigen. Insbesondere das individuelle Risiko eines Interaktionspotentials mit anderen CYT-P-450 abhängigen Substanzen (z. B. Fentanyl) ist dabei zu beachten [61].

Ein individualisiertes postoperatives Schmerzmanagement sollte bereits präoperativ festgelegt werden. Für die Auswahl geeigneter supportiver Substanzen und Strategien sei auf die AWMF S3-Leitlinie „Behandlung akuter perioperativer und posttraumatischer Schmerzen verwiesen“ [31]. Weitere Empfehlungen für die adäquate perioperative Analgesie sind im allgemeinen Teil und den prozedurenspezifischen Empfehlungen dieser Leitlinie sowie der Leitlinie „Perioperatives Management gastrointestinaler Tumoren (POMGAT)“ enthalten [28].

##### A.2.4.2 Substanzabhängigkeit und Substitution.

Generell sollte bei Substanzabhängigkeit zur Risikoreduktion perioperativ der Substanzkonsum nach Möglichkeit fortgesetzt (z. B. Alkohol, Nikotin) oder substituiert (z. B. Opioide) werden, um perioperative Unruhe, Entzugssymptome und lebensbedrohliche vegetative Entgleisungen zu vermeiden. Neben Alkohol, Nikotin und Opioide sind hierbei insbesondere Benzodiazepine, Gabapentinoide, Cannabinoide und Z‑Substanzen von Bedeutung. Die bereits vorbestehende Opioideinnahme ist im Rahmen der präoperativen Risikoevaluation aufgrund einer möglichen Abhängigkeit, Toleranzentwicklung und psychosozialer Krankheitsaspekte von hoher Relevanz. Die Opioid-Abhängigkeit ist eine chronische Erkrankung mit wechselnden Phasen mit und ohne Konsum bzw. Substitution. Von zentraler Bedeutung ist bei diesen sehr Stress-reagiblen Patientinnen und Patienten eine vertrauensvolle und enge Begleitung in der perioperativen Phase.

## B. Erweiterte Diagnostik

In Abhängigkeit vorbestehender Komorbiditäten können folgende weiterführende diagnostische Maßnahmen ergriffen werden:

### B.1 Allgemeine Untersuchungen

#### B.1.1 Blutuntersuchungen

Die präoperative Labor-Diagnostik hat zum Ziel, auffällige Befunde in der Anamnese und/oder der körperlichen Untersuchung zu überprüfen und den Schweregrad bestehender Erkrankungen abzuschätzen. Mit zunehmender Anzahl von Laborparametern steigt jedoch auch die Wahrscheinlichkeit, zufällig außerhalb der ‚Norm‘ liegende Parameter zu entdecken. Eine routinemäßige Erhebung von Laboruntersuchungen (‚Screening‘) soll nicht durchgeführt werden [62] ^**E21**^. Auch die Schwere des Eingriffs oder das Alter (s. unten) stellen per se keine gesicherten Indikationen zur präoperativen Bestimmung von Laborparametern dar.

Dies gilt auch für die Bestimmung von Parametern der Blutgerinnung [63]. So ist die konventionelle Gerinnungsdiagnostik (aPTT, INR, Thrombozytenzahl) nicht in der Lage, die häufigsten Störungen der Blutgerinnung (angeborene und erworbene Störungen der Thrombozytenfunktion sowie des von-Willebrand-Faktors) zu erfassen. Eine Gerinnungsdiagnostik wird daher nur empfohlen bei entsprechender Medikamentenanamnese (z. B. Einnahme oraler Vit-K-Antagonisten) sowie bei anamnestischem Verdacht auf eine Gerinnungsstörung (positive Blutungsanamnese auf der Basis eines standardisierten Fragebogens [64]).

Bei Patientinnen und Patienten mit bekannten oder vermuteten Organerkrankungen soll die Bestimmung der in Tab. 13 dargestellten Laborparameter im Blut als sinnvoller Minimalstandard durchgeführt werden ^**E22**^.

**Table 13:** 

	Organerkrankung			
Parameter	Herz/Lunge	Leber	Niere	Blut
Hämoglobin	+	+	+	+
Leukozyten	–	–	–	+
Thrombozyten	–	+	–	+
Natrium, Kalium	+	+	+	+
Kreatinin	+	+	+	+
ASAT; Bilirubin, aPTT und INR	–	+	–	–

*ASAT* Aspartat-Aminotransferase, *aPTT* aktivierte partielle Thromboplastinzeit, *INR* International Normalized Ratio

Die Bestimmung der Hämoglobinkonzentration ist unabhängig von vorbestehenden Organerkrankungen zu empfehlen, wenn der geplante Eingriff ein relevantes Blutungsrisiko aufweist (> 10 % Risiko für Bluttransfusionen).

Ein Diabetes mellitus ist ein relevanter perioperativer Risikofaktor und kann trotz sorgfältiger Anamnese und körperlicher Untersuchung präoperativ unentdeckt bleiben. Ob die routinemäßige präoperative Kontrolle des Blutzuckers bei klinisch unauffälligen Patientinnen und Patienten das perioperative Risiko reduzieren kann, ist jedoch unklar. Eine präoperative Bestimmung des Nüchtern-Blutzuckers wird daher nur vor Operationen mit hohem eingriffsbezogenen Risiko, bei Vorliegen weiterer kardialer Risikofaktoren sowie bei Patientinnen und Patienten mit Adipositas (Body Mass Index (BMI) > 30 kg/m^2^) empfohlen [65]. Bei bekanntem oder neu diagnostiziertem Diabetes mellitus sollte der Blutzucker perioperativ engmaschig kontrolliert und eingestellt werden.

Weitergehende Laboranalysen sollten individualisiert auf der Basis von Anamnese und körperlichem Untersuchungsbefund nur dann erfolgen, wenn sie absehbar das perioperative Vorgehen beeinflussen. Alleine das Serum-Kreatinin sollte, unter Berücksichtigung alters- und geschlechtsabhängiger Veränderungen, bei Operationen mit hohem eingriffsbezogenen Risiko bekannt sein [19] ^**E23**^ (Tab. 14).

**Table 14:** 

	Empfehlung	Empfehlungsgrad	
E21	Die routinemäßige Blutuntersuchung SOLL NICHT durchgeführt werden.	A	⇓⇓
E22	Bei bekannten Organerkrankungen SOLL ein Minimalstandard der Blutuntersuchung durchgeführt werden.	A	⇑⇑
E23	Das Serum-Kreatinin SOLLTE bei Operationen mit hohem eingriffsbezogenen Risiko bekannt sein.	B	⇑

#### B.1.2 Kardiale Biomarker

Die präoperative Bestimmung von kardialen Biomarkern, welche eine myokardiale Schädigung (hoch sensitives kardiales Troponin T/I – Hs-cTn T/I) quantifizieren sowie Rückschlüsse auf die hämodynamische Einschränkung mit konsekutiv erhöhter kardialer Wandspannung (B-Typ natriuretisches Peptid/NT-proBNP) ermöglichen, haben an Bedeutung gewonnen. So konnte in großen prospektiven Studien gezeigt werden, dass sowohl Hs-cTn T/I als auch BNP/NT-proBNP eine hohe prognostische Bedeutung für perioperative Komplikationen einschließlich kardiovaskulärer Tod, Herzstillstand, akute Herzinsuffizienz und Tachyarrhythmie haben [66–70]. Unter Berücksichtigung eines Grenzwertes von 14 ng/L für hsTnT und 300 pg/mL für NT-proBNP konnte ein Letalitätsunterschied von 6,9 vs. 1,2 % (hsTnT) und 4,8 vs. 1,4 % (NT-proBNP) bei Patientinnen und Patienten, die sich elektiven, nicht-herzchirurgischen Eingriffen unterziehen mussten, nachgewiesen werden [61]. Aus diesem Grund hat in der Leitlinie der ESC die Bestimmung von hoch sensitivem kardialen Troponin T/I – Hs-cTn T/I vor, 24 und 48 h nach elektiven operativen Eingriffen mit mittlerem und hohem Risiko eine Klasse I Empfehlung erhalten [5, 6]. In einer Leitlinie der Europäischen Gesellschaft für Anästhesiologie und Intensivmedizin (ESAIC) zum Einsatz von kardialen Biomarkern wird zwischen der Verwendung des hoch sensitiven kardialen Troponin (isoliert vor oder kombiniert vor und nach einem operativen Eingriff) als Prognosefaktor, zur Einschätzung des individuellen Risikos oder zur Steuerung einer die Patientinnen und Patienten präoperativ vorbereitenden Therapie unterschieden [7]. In dieser Leitlinie wird lediglich für die Verwendung von Troponin als Prognosefaktor eine schwache Empfehlung ausgesprochen. Die Autoren der vorliegenden Handlungsempfehlungen kommen zu dem Schluss, dass unter Berücksichtigung der aktuellen Literatur und Europäischen Empfehlungen (ESC und ESAIC) [5–7] bei Patientinnen und Patienten mit manifester kardiovaskulärer Erkrankung, kardiovaskulären Risikofaktoren oder kardiovaskulären Symptomen, die sich einer Operation mit mittlerem und hohem eingriffsbezogenen Risiko unterziehen, eine präoperative und 24/48 h postoperative Bestimmung von Hs-cTn T/I durchgeführt werden sollte ^**E24**^. Dies kann, wie im Kapitel A.1.1.4 beschrieben, in eine weiterführenden nicht-invasiven oder invasiven Diagnostik, therapeutischen Intervention oder intensivierten intra- und postoperativen Überwachung resultieren. Potenziell negative Konsequenzen durch zusätzliche Untersuchungen und die damit verbundene Verzögerung des Eingriffs sollen hierbei berücksichtigt und interdisziplinär bewertet werden.

BNP/NT-proBNP stellt einen quantitativen Marker bei der Diagnose der Herzinsuffizienz dar [5, 6, 71]. BNP oder NT-proBNP sollen nach Empfehlung der ESC vor sowie 24 und 48 h nach Eingriffen mit mittlerem und hohem Risiko durchgeführt werden (IIa). In der Empfehlung der ESAIC wird für die Bestimmung von BNP oder NT-proBNP (vor elektiven Eingriffen) als Prognosefaktor eine schwache Empfehlung ausgesprochen [7]. Keine Empfehlung erhält die isolierte, postoperative Bestimmung von BNP/NT-proBNP. Die Autoren der vorliegenden Handlungsempfehlungen kommen zu dem Schluss, dass unter Berücksichtigung der aktuellen Literatur und Empfehlungen (ESC und ESAIC) [5, 6, 64] bei Patientinnen und Patienten mit bekannter kardiovaskulärer Erkrankung oder kardiovaskulären Risikofaktoren oder Symptomen, die auf eine kardiovaskuläre Erkrankung hinweisen, präoperativ bei Operationen mit mittlerem oder hohem eingriffsbezogenen Risiko eine BNP/NT-proBNP-Diagnostik erwogen werden kann ^**E25**^. Bei Patientinnen und Patienten mit niedrigem Risiko, die sich einer Operation mit niedrigem oder mittlerem eingriffsbezogenen Risiko unterziehen, soll die Bestimmung des Hs-cTn T/I oder BNP/NT-proBNP nicht durchgeführt werden ^**E26**^ (Tab. 15).

**Table 15:** 

	Empfehlung	Empfehlungsgrad	
E24	Bei PatientInnen mit präoperativ bekannten kardiovaskulären Risiken, Symptomen oder Erkrankungen SOLLTE die Bestimmung des „High-sensitivity cardiac troponin T/I (Hs-cTn T/I)“ präoperativ und 24/48 h postoperativ bei elektiven Operationen mit mittlerem und hohem eingriffsbezogenen Risiko durchgeführt werden.	B	⇑
E25	Bei PatientInnen mit bekannten präoperativ kardiovaskulären Risiken, Symptomen oder Erkrankungen KANN die Bestimmung des „BNP“ oder „NT-proBNP“ präoperativ vor Operationen mit mittlerem und hohem eingriffsbezogenen Risiko erwogen werden.	0	⇔
E26	Bei PatientInnen mit niedrigem Risiko, die sich einer elektiven Operation mit niedrigem oder mittlerem eingriffsbezogenen Risiko unterziehen, SOLL die Bestimmung des Hs-cTn T/I oder BNP/NT-proBNP NICHT durchgeführt werden [5, 6, 10].	A	⇓⇓

*Hs-cTn T/I* High-sensitivity cardiac troponin T/I, *BNP* brain natriuretic peptide, *NT-proBNP* N-terminal pro b‑type natriuretic peptide

### B.2 Diagnostik bei Patientinnen und Patienten mit kardiovaskulären Vorerkrankungen

Bei Patientinnen und Patienten mit bekannten oder vermuteten kardiovaskulären Vorerkrankungen kann präoperativ eine differenzierte kardiologische Abklärung erforderlich sein. Die Indikation hierfür ist allerdings wegen des vielfach unklaren Nutzens vorangestellter operativer, kardiologischer oder herzchirurgischer Interventionen in Hinblick auf die perioperative Morbidität und Letalität streng zu stellen. Die rationale Abschätzung des perioperativen kardialen Risikos sowie die Entscheidung für oder gegen eine erweiterte präoperative Diagnostik basiert dabei auf den in Abschnitt A.2.1 genannten Faktoren. Grundsätzlich gilt, dass bei akut-symptomatischen Herzerkrankungen („active cardiac condition“) nur Notfall-Operationen durchgeführt werden sollen.

#### B.2.1 12-Kanal EKG

Das präoperative 12-Kanal EKG ist eine einfach und kostengünstig durchzuführende Untersuchung, die bisher unbekannte Herzrhythmusstörungen vor operativen Eingriffen ermitteln und den Vergleich mit vorangegangenen EKG-Untersuchungen oder intra- oder postoperativ durchgeführten EKG-Untersuchungen ermöglichen [5, 6]. Für Die Indikationsstellung erscheinen folgende Empfehlungen sinnvoll:

- Bei anamnestisch unauffälligen und Patientinnen und Patienten vor einer Operation mit niedrigem eingriffsbezogenen Risiko sind anästhesierelevante Befunde selten. Ein präoperatives 12-Kanal EKG ist hier – unabhängig vom Alter – nicht erforderlich [72]
- Bei kardial asymptomatischen Patientinnen und Patienten, aber anamnestisch eruierbaren kardialen Risikofaktoren sollte ein 12-Kanal EKG vor Operationen mit mittlerem oder hohem eingriffsbezogenen Risiko abgeleitet werden [73]
- Bei Patientinnen und Patienten mit klinischen Symptomen einer ischämischen Herzerkrankung, bei Herzrhythmusstörungen, Klappenerkrankungen, Herzvitien oder einer (Links- bzw. Rechts‑) Herzinsuffizienz oder bei Trägern eines implantierten Defibrillators (ICD) soll ein präoperatives EKG abgeleitet werden ^**E27**^

Bei Trägern eines Herzschrittmachers ist ein präoperatives EKG nicht erforderlich, sofern die regelmäßig vorgesehenen Schrittmacherkontrolltermine eingehalten wurden und der Patient keine klinischen Symptome aufweist (Tab. 16).

**Table 16:** 

	Empfehlung	Empfehlungsgrad	
E27	Bei PatientInnen mit bekannter kardiovaskulärer Erkrankung oder mindestens einem kardiovaskulären Risikofaktor oder Symptomen bzw. Befunden, die auf eine kardiovaskuläre Erkrankung hinweisen, SOLLTE präoperativ vor Operationen mit mittlerem und hohem eingriffsbezogenen Risiko ein 12-Kanal EKG durchgeführt werden [5, 6, 10].	B	⇑

#### B.2.2 (Doppler-)Sonographie 

##### B.2.2.1 Transthorakale Doppler-Echokardiographie.

Die Transthorakale (Doppler-)Echokardiographie (TTE) in Ruhe erlaubt die direkte Beurteilung von Größe, Geometrie und Funktion der Herzkammern sowie der intrakardialen Strömungsverhältnisse. Häufigste Indikationen präoperativ sind:

- die Beurteilung der rechts- und linksventrikulären Pumpfunktion sowie
- die Beurteilung von möglichen Herzvitien und Herzklappendefekten bei Patientinnen und Patienten mit Zeichen einer Herzinsuffizienz oder mit pathologischen Herzgeräuschen

In einer großen retrospektiven Untersuchung konnte nicht gezeigt werden, dass die routinemäßige TTE vor Hoch-Risiko-Eingriffen die postoperative MACE Rate senkt [74]. Daher soll eine TTE nur bei Patientinnen und Patienten mit deutlich eingeschränkter Belastbarkeit und/oder erhöhtem BNP/NT-proBNP oder neu aufgetretenem Herzgeräusch vor Eingriffen mit hohem eingriffsbezogenen Risiko durchgeführt werden [5, 6, 75, 76]. Eine TTE soll grundsätzlich bei allen Patientinnen und Patienten mit neu aufgetretenem Herzgeräusch und Symptomen kardiovaskulärer Erkrankungen oder bei Dyspnoe und/oder peripheren Oedemen und erhöhtem NT-proBNP/BNP vor Operationen durchgeführt werden ^**E28**^. Eine stabile Herzinsuffizienz oder eine vermutete bzw. nachgewiesene ischämische Herzerkrankung (KHK) allein sollten nicht durch eine präoperative Echokardiographie abgeklärt werden ^**E29**^. Auch die Routineevaluation der linksventrikulären Funktion soll nicht durchgeführt werden ^**E30**^. Die TTE kann bei Patientinnen und Patienten mit eingeschränkter kardiovaskulärer Funktion, pathologischem EKG, hohem NT-proBNP/BNP oder mindestens einem kardialem Risikofaktor vor Operationen mit mittlerem eingriffsbezogenen Risiko erwogen werden ^**E31**^ (Tab. 17).

**Table 17:** 

	Empfehlung	Empfehlungsgrad	
E28	Die TTE SOLL bei PatientInnen mit neu aufgetretenem Herzgeräusch und Symptomen kardiovaskulärer Erkrankungen oder bei Dyspnoe und/oder peripheren Oedemen und hohem NT-proBNP/BNP vor Operationen durchgeführt werden [5, 6, 10].	A	⇑⇑
E29	Eine stabile Herzinsuffizienz oder eine vermutete bzw. nachgewiesene ischämische Herzerkrankung (KHK) SOLLTEN NICHT allein durch eine präoperative Echokardiographie abgeklärt werden [5, 6, 10].	B	⇓
E30	Die Routineevaluation der linksventrikulären Funktion SOLL NICHT durchgeführt werden [5, 6, 10].	A	⇓⇓
E31	Die TTE KANN bei PatientInnen mit eingeschränkter kardiovaskulärer Funktion, pathologischem EKG, hohem NT-proBNP/BNP oder mindestens einem kardialen Risikofaktor vor Operationen mit mittlerem eingriffsbezogenen Risiko erwogen werden [5, 6, 10].	0	⇔

*TTE* Transthorakale Doppler Echokardiographie, *NT-proBNP* N-terminal pro b‑type natriuretic peptide, *BNP* Brain natriuretic peptide

##### B.2.2.2 Doppler-Sonographie der Halsgefäße.

Eine Reihe von operativen Eingriffen (z. B. Operationen im Kopf/Hals-Bereich, Hüftoperationen, Notfalloperationen) gehen perioperativ mit einer erhöhten Inzidenz apoplektischer, meist ischämischer Insulte einher. Die intraoperative, auch lagerungsbedingte Hypotension (z. B. im Rahmen halbsitzender Lagerung) gilt als einer der wesentlichen prädisponierenden Faktoren [77]. Bei Patientinnen und Patienten mit asymptomatischen oder symptomatischen Verschlüssen der Aa. carotides gelten die Behandlungskriterien der ESC Guidelines zur Diagnose und Behandlung peripher arterieller Verschlusskrankheiten [14]. Patientinnen und Patienten, die in den vergangenen 6 Monaten Symptome hatten, die auf eine Stenose der Aa. carotides hinweisen, SOLLTEN präoperativ einer Diagnostik mittels Sonographie zugeführt werden [78] ^**E32**^. Bei Patientinnen und Patienten mit symptomatischen Verschlüssen der Aa. carotides kann sich eine Revaskularisierung der Gefäße vor einem elektiven Eingriff für die Patientin bzw. den Patienten als vorteilhaft erweisen, wenn eine TIA oder ein Apoplektischer Insult in den vorvergangenen 3 Monaten und eine über 70 %ige Stenose der Aa. carotides nachgewiesen wurden. Die Entscheidung zur Revaskularisierung der Gefäße sollte in dieser Konstellation vor einem elektiven, nicht herz-thoraxchirurgischen Eingriff getroffen werden [5, 6, 79]. Bei Patientinnen und Patienten mit ausgedehnten, asymptomatischen Verschlüssen der Aa. carotides sollten die gleichen Indikationen zur Revaskularisierung wie für Patientinnen und Patienten ohne operativen Eingriff gelten [5, 6] ^**E33**^ (Tab. 18).

**Table 18:** 

	Empfehlung	Empfehlungsgrad	
E32	PatientInnen, die in den letzten 6 Monaten Symptome hatten, die auf eine Stenose der Aa. carotides hinweisen, SOLLTEN präoperativ einer Diagnostik (Sonographie) zugeführt werden.	B	⇑
E33	Für PatientInnen mit Verschluss der Aa. carotides, die sich einer elektiven Operation unterziehen müssen, SOLLTEN die gleichen Indikationen zur Revaskularisierung wie für PatientInnen ohne operativen Eingriff gelten.	B	⇑

#### B.2.3 Erweiterte kardiologische Diagnostik

##### B.2.3.1 Nichtinvasive Untersuchungen zur Diagnostik einer myokardialen Ischämie.

Ein Belastungs-EKG kann in seltenen Fällen zur klinischen Beurteilung der Belastbarkeit herangezogen werden. Zum Nachweis einer myokardialen Ischämie hat das Belastungs-EKG aber nur dann einen Stellenwert, wenn bildgebende Verfahren zur Beurteilung einer myokardialen Ischämie nicht zur Verfügung stehen [5, 6, 80].

Die lokalisierte Ischämiediagnostik (Stressechokardiographie, Myokardszintigraphie, Stress-MRT) ist kein Bestandteil der routinemäßigen präoperativen kardiovaskulären Risikoevaluation ^**E34**^ [5, 6]. Die Wahl des Verfahrens richtet sich hierbei nach den lokalen Gegebenheiten und unter Berücksichtigung der aktuellen Leitlinien und Empfehlungen [80–82]. Zur präoperativen Risikoevaluation liefern die Stressechokardiographie und die Myokardszinitgraphie vergleichbare Ergebnisse [83]. Der Nachweis einer relevanten myokardialen Ischämie in der Myokardszintigraphie (> 10 % des linksventrikulären Myokards betreffend) ist ein erwiesener Prädiktor für das Auftreten relevanter kardiovaskulärer Ereignisse (MACE) [84]. Daher sollen bildgebende Verfahren zur Bestimmung der myokardialen Ischämie vor Operationen mit hohem eingriffsbezogenen Risiko bei Patientinnen und Patienten mit eingeschränkter Belastbarkeit und hoher Wahrscheinlichkeit für das Vorliegen einer koronaren Herzerkrankung eingesetzt werden ^**E35**^. Darüber hinaus sollte die bildgebende Ischämiediagnostik auch bei asymptomatischen Patientinnen und Patienten vor Operationen mit hohem eingriffsbezogenen Risiko und eingeschränkter körperlicher Belastbarkeit sowie stattgehabter PCI oder ACB-Operation durchgeführt werden ^**E36**^ (Tab. 19).

**Table 19:** 

	Empfehlung	Empfehlungsgrad	
E34	Eine bildgebende Ischämiediagnostik SOLL routinemäßig präoperativ NICHT durchgeführt werden.	A	⇓⇓
E35	Eine bildgebende Ischämiediagnostik SOLL vor Operationen mit hohem eingriffsbezogenen Risiko und eingeschränkter Belastbarkeit und hoher Wahrscheinlichkeit für das Vorliegen einer koronaren Herzerkrankung erfolgen [5, 6].	A	⇑⇑
E36	Eine bildgebende Ischämiediagnostik SOLLTE vor Operationen mit hohem eingriffsbezogenen Risiko bei asymptomatischen PatientInnen, eingeschränkter Belastbarkeit und vorangegangener PCI bzw. ACB-Operation durchgeführt werden [5, 6].	B	⇑

*PCI* perkutane Koronarintervention, *ACB* aortokoronare Bypassoperation

##### B.2.3.2 Nichtinvasive (Kardio-CT) und invasive Koronardiagnostik (Koronarangiographie).

*B.2.3.2.1 Koronar-CT-Angiographie (CCTA).* Zu Indikation und Stellenwert von Kardio-MRT und speziell der Koronar-CT-Angiographie (CCTA) im Rahmen der präoperativen Evaluation wurden zahlreiche klinische Untersuchungen durchgeführt [85, 86]. Die CCTA wird empfohlen als eine Alternative zur invasiven Koronarangiographie bei Beurteilung eines möglichen chronischen Koronarsyndroms (CCS) bei normalem EKG und normalen kardialen Troponin-Werten [5, 6, 13]. Im Weiteren kann die CCTA bei Patientinnen und Patienten mit niedriger bzw. intermediärer Wahrscheinlichkeit für das Vorliegen eines CCS in Betracht gezogen werden. Sie ist für Patientinnen und Patienten geeignet, bei denen ein Belastungstest nicht möglich ist ^**E37**^.

*B.2.3.2.2 Invasive Koronardiagnostik (Koronarangiographie).* Eine invasive Koronardiagnostik (‚Herzkatheter‘) ist bei Patientinnen und Patienten vor elektiven Operationen nur sehr selten zur Abschätzung des perioperativen Risikos indiziert [5, 6]. Grundsätzlich entspricht dabei die Indikation derjenigen für die Durchführung einer Koronarangiographie bzw. einer PCI unabhängig von einer bevorstehenden Operation ^**E38**^. Die präoperative Koronarangiographie kann aber auch bei Patientinnen und Patienten mit chronischem Koronarsyndrom, die sich einer elektiven asymptomatischen Karotis-Endarterektomie unterziehen müssen, erwogen werden [5, 6] ^**E39**^. Bei Patientinnen und Patienten mit stabilem chronischen Koronarsyndrom soll eine routinemäßige präoperative Koronarangiographie nicht indiziert werden ^**E40**^ (Tab. 20).

**Table 20:** 

	Empfehlung	Empfehlungsgrad	
E37	Die Kardio-CT-Diagnostik KANN bei PatientInnen mit niedrigem bzw. intermediärem Risiko für das Vorliegen eines chronischen Koronarsyndroms oder bei PatientInnen, die ungeeignet sind für bildgebende Belastungstests, vor Operationen mit mittlerem bis hohem eingriffsbezogenen Risiko als Ausschluss-Diagnostik erwogen werden [5, 6].	0	⇔
E38	Die Indikation für die invasive Koronardiagnostik soll präoperativ nach den gleichen Empfehlungen erfolgen wie im nichtoperativen Bereich [5, 6, 10].	A	⇑⇑
E39	Die präoperative Koronarangiographie KANN bei PatientInnen mit chronischem Koronarsyndrom, die sich einer elektiven asymptomatischen Karotis-Endarterektomie unterziehen müssen, erwogen werden [5, 6, 10].	0	⇔
E40	Bei PatientInnen mit stabilem chronischen Koronarsyndrom SOLL eine routinemäßige präoperative Koronarangiographie für Operationen mit niedrigem oder mittlerem eingriffsbezogenen Risiko NICHT indiziert werden [5, 6, 10].	A	⇓⇓

### B.3 Diagnostik bei Patientinnen und Patienten mit pulmonalen Vorerkrankungen

#### B.3.1 Röntgenuntersuchungen der Thoraxorgane (Thoraxröntgen)

Routinemäßige präoperative Thoraxröntgen-Aufnahmen (Screening) können nicht empfohlen werden, da sie das perioperative Management in der Regel nicht verändern [43, 87, 88] und bei asymptomatischen Patientinnen und Patienten in der Regel kein Nutzen zu erwarten ist [42]. Röntgenaufnahmen des Thorax sind daher ausschließlich gezielten Fragestellungen vorbehalten [88] und nur indiziert, wenn eine klinische Verdachtsdiagnose mit Konsequenzen für das perioperative Vorgehen (z. B. Pleuraerguss, Atelektase, Pneumonie) erhärtet oder ausgeschlossen werden sollen (Abb. 2; [89]). Daneben kann eine Thoraxröntgen-Aufnahme in speziellen Fällen auch unabhängig von kardiopulmonalen Symptomen für die OP- und/oder Anästhesieplanung sinnvoll sein (z. B. zur Abschätzung einer Trachealverlagerung bei Struma). Der diagnostische Nutzen muss sorgsam gegenüber dem Aufwand und der Strahlenbelastung abgewogen werden [42]. Der Nutzen fester Altersgrenzen für die routinemäßige Anfertigung einer Thoraxröntgenaufnahme ist wissenschaftlich nicht belegt.

#### B.3.2 Lungenfunktionsuntersuchungen

An apparativen Verfahren zur Evaluation der Lungenfunktion stehen die Messung der arteriellen Sauerstoffsättigung mithilfe der Pulsoximetrie (in Ruhe bzw. unter Belastung), die Spirometrie bzw. Spiroergometrie, die Body-Plethysmographie sowie die arterielle Blutgasanalytik zur Verfügung.

Generell ist eine spezielle Risikoevaluation mittels Lungenfunktionsdiagnostik präoperative nur sinnvoll, wenn sie die Vorhersage von perioperativ pulmonalen Komplikationen verbessert [41, 90, 91]. Bei lungengesunden Patientinnen und Patienten sollen apparative Verfahren zur pulmonalen Diagnostik routinemäßig nicht zum Einsatz kommen ^**E41**^. Röntgenaufnahmen des Thorax sollen ausschließlich bei Patientinnen und Patienten mit pulmonalen Verdachtsdiagnosen, die das perioperative Vorgehen verändern, durchgeführt werden ^**E42**^. Ein diagnostischer Mehrwert einer präoperativen Spirometrie und BGA bezüglich der Vorhersage von perioperativ pulmonalen Komplikationen ist nicht belegt. Präoperative pulmonale Funktionstests werden daher vor nicht herz-thoraxchirurgischen Eingriffen nicht routinemäßig empfohlen [91]. Eine Spirometrie kann bei Patientinnen und Patienten mit typischen Zeichen für eine COPD vor Oberbaucheingriffen erwogen werden [91]. Allerdings verbessern pulmonale Funktionstests die Vorhersage von perioperativ pulmonalen Komplikationen selbst bei Patientinnen und Patienten mit bekannter oder vermuteter COPD vor großen nicht herzchirurgischen Eingriffen nicht ([90]; Abb. 2). Pulmonale Funktionstests können allerdings unabhängig von der geplanten Operation sinnvoll sein (z. B. Diagnosesicherung einer COPD). Sie können in diesem Fall aber auch postoperativ im Verlauf durchgeführt werden (Abb. 2; Tab. 21).

**Table 21:** 

	Empfehlung	Empfehlungsgrad	
E41	Bei lungengesunden PatientInnen SOLLEN apparative Verfahren zur pulmonalen Diagnostik routinemäßig NICHT zum Einsatz kommen.	A	⇓⇓
E42	Röntgenaufnahmen des Thorax SOLLEN bei PatientInnen mit pulmonalen Verdachtsdiagnosen, die das perioperative Vorgehen verändern, durchgeführt werden.	A	⇑⇑

## C. Präoperativer Umgang mit der Dauermedikation

Die Erfassung der patientinnen- und patienteneigenen Dauermedikation ist wesentlicher Bestandteil der präoperativen Evaluation. Ob es medizinisch sinnvoll ist, eine präoperativ bestehende Medikation perioperativ weiterzuführen oder bestimmte Medikamente vor einer Operation neu anzusetzen, ist vielfach nur unzureichend untersucht. Die folgenden Empfehlungen sind daher nur ein Anhalt und bedürfen der kritischen Überprüfung und gegebenenfalls Ergänzung in jedem Einzelfall.

### C.1 Kreislaufwirksame Pharmaka

Eine antianginöse, antihypertensive oder antiarrhythmische Therapie sollte in aller Regel perioperativ fortgeführt werden. Dies gilt besonders für β‑Rezeptorantagonisten und Nitrate, da hier ein Absetzen der Therapie eine Myokardischämie mit Myokardinfarkt auslösen kann.

#### C.1.1 Dauermedikation mit Betablockern

Die Indikation zum präoperativen Neubeginn einer Therapie mit β‑Rezeptorantagonisten wird kontrovers diskutiert, aber zum jetzigen Zeitpunkt nicht empfohlen [92]. Die Inzidenz für den perioperativen Tod, Schlaganfall oder eine klinisch signifikante Hypotension oder Bradykardie ist bei Patientinnen und Patienten mit neu begonnener β‑Rezeptorantagonisten Therapie erhöht [93, 94]. Die präoperativ erstmalige Gabe eines β‑Rezeptorantagonisten kann aber erwogen werden

- bei allen Patientinnen und Patienten mit 2 oder mehr kardialen Risikofaktoren nach Lee (RCRI) oder einer ASA-Klasse von ≥ 3, die sich einer Operation mit hohem eingriffsbezogenen Risiko unterziehen [95] ^**E43**^
  sowie
- unabhängig von der Art des Eingriffs bei allen Patientinnen und Patienten mit nachgewiesener KHK und dokumentierter Myokardischämie unter Belastung [73, 96] ^**E44**^.

Eine präoperative Neueinstellung soll nicht durchgeführt werden, wenn eine Dosistitration des β‑Rezeptorantagonisten nach Herzfrequenz und Blutdruck mit ausreichendem Abstand zur Operation nicht gewährleistet ist oder lediglich eine Operation mit niedrigem eingriffsbezogenen Risiko geplant ist [73] ^**E45**^.

Eine vorbestehende Dauermedikation mit β‑Rezeptorantagonisten soll präoperativ fortgeführt werden ^**E46**^, so führte das präoperative Absetzen der Medikation in großen Beobachtungsstudien zu einer steigenden Letalität und erhöhte das Risiko für Herzrhythmusstörungen [5, 6, 95, 96].

#### C.1.2 Ca^2+^-Antagonisten

Ob *Ca*^*2+*^*-Antagonisten* (Calciumkanalblocker) das perioperative Behandlungsergebnis verbessern, ist unklar. In einer Meta-Analyse aus 11 randomisierten Studien führte die Behandlung mit Calciumkanalblockern postoperativ zu einer Reduktion an myokardialen Ischämien und supraventrikulären Tachykardien [97]. Im Allgemeinen wird eine bestehende Dauertherapie perioperativ weitergeführt. Daher ist aus Sicht der Autoren die Fortführung einer Dauertherapie mit Calciumkanalblockern zu empfehlen.

#### C.1.3 Diuretika

Diuretika werden häufig zur Therapie der Hypertonie oder Herzinsuffizienz eingesetzt. Die Unterbrechung der Gabe von Diuretika am Operationstag kann erwogen werden ^**E47**^. Die Fortführung der Therapie mit Diuretika am Tag der Operation birgt das Risiko der perioperativen Hypovolämie oder Hypokaliämie. Insbesondere bei Patientinnen und Patienten mit Herzinsuffizienz sollte eine Dosisanpassung erfolgen [5, 6] und eine Dauertherapie mit Diuretika unmittelbar postoperativ wiederaufgenommen werden [98].

#### C.1.4 Angiotensin Converting Enzyme-Hemmer/Angiotensin-II-Rezeptorantagonisten

Die Fortführung der präoperativen Gabe von Angiotensin Converting Enzyme (ACE)-Hemmer (ACEI) oder Angiotensin-II-Rezeptorantagonisten (ARB) wird kontrovers diskutiert. Bei Patientinnen und Patienten, die ACEI oder ARB am Operationstag einnehmen, wurden intraoperativ gehäuft hypotensive Phasen detektiert, die nur durch die Gabe von Vasopressin-Analoga erfolgreich behandelt werden konnten [99]. In einer großen prospektiven multizentrischen Kohortenstudie wurde durch eine 24-stündige, präoperative ACEI/ARB-Pause das Risiko für Tod, Schlaganfall oder Myokardinfarkt signifikant gesenkt [100]. Ein Zusammenhang zwischen der Weitergabe von ACEI/ARB und dem Auftreten eines postoperativen Nierenversagens nach großen abdominalchirurgischen Eingriffen konnte nicht nachgewiesen werden.

Vor dem Hintergrund der aktuellen Evidenzlage sollten ACEI/ARB, die zur Therapie der arteriellen Hypertonie eingesetzt werden, vorübergehend (am Morgen der Operation) pausiert werden ^**E48**^. Bei Patientinnen und Patienten, die eine vorbestehende Dysfunktion der linksventrikulären Funktion haben, sollten ACEI/ARB jedoch weitergeführt werden. Bei Operationen mit niedrigem eingriffsbezogenen Risiko, kurzer OP-Dauer und geringen Volumenverschiebungen kann eine Fortführung der ACE-Hemmer/AT-II-Blocker-Therapie ungeachtet der Indikation sinnvoll sein. Hier ist eine Einzelfallentscheidung für die jeweilige Patientin bzw. den jeweiligen Patienten in Kenntnis der Blutdruckwerte bzw. dem Ausmaß der Herzinsuffizienz durch das interdisziplinäre behandelnde Team zu treffen.

#### C.1.5 Angiotensin-Rezeptor-Neprilysin-Inhibitoren und I_f_*-Kanal-Inhibitoren

Angiotensin-Rezeptor-Neprilysin-Inhibitoren (ARNI) vereinen als neue Medikamentengruppe die Eigenschaften von ACE-Hemmern und Neprilysin-Inhibitoren. Durch die Hemmung des Enzyms Neprilysin wird der Abbau u. a. von Bradykinin und natriuretischem Peptid verlangsamt, was wiederum zu einer verstärkten Diurese und Natriurese führt. Darüber hinaus wird die myokardiale Relaxation verbessert und ein Umbau des Myokards im Rahmen einer Herzinsuffizienz verlangsamt. ARNI werden eingesetzt bei Patientinnen und Patienten mit unter ACE-Hemmer Therapie noch symptomatischer Herzinsuffizienz und einer LVEF < 35 %. Zu Beginn der Therapie kann es hier zu teilweise erheblichen Hypotonien kommen.

Der I_f_-Kanal-Inhibitor Ivabradin ist ein negativ chronotropes Medikament mit gering hypotensiven Eigenschaften [5, 6]. Ivabradin wird bei Patientinnen und Patienten mit chronischer Herzinsuffizienz eingesetzt, die trotz optimaler medikamentöser Therapie symptomatisch und sinustachykard sind. Die Bedeutung dieser Medikamente in der perioperativen Phase ist noch unklar. Vor dem Hintergrund der Therapieindikation erscheint eine Fortführung jedoch sinnvoll [101].

#### C.1.6 Digitalis-Glykoside

Digitalis-Glykoside zur Therapie einer chronischen Herzinsuffizienz werden wegen ihrer geringen therapeutischen Breite, schlechten Steuerbarkeit und arrhythmogenen Potenz oft präoperativ abgesetzt. Angesichts der langen Halbwertszeit der Präparate ist der Nutzen eines kurzfristigen Absetzens jedoch unsicher. Patientinnen und Patienten mit normofrequenter absoluter Arrhythmie sollten die Medikamente weiter erhalten, da hier das Absetzen perioperativ Tachyarrhythmien auslösen kann. Da auch die Indikation für den Einsatz von Digitalis-Glykosiden bei chronischer Herzinsuffizienz auf die Frequenzkontrolle bei gleichzeitig bestehendem Vorhofflimmern beschränkt ist, sollte die Medikation auch in diesem Fall fortgesetzt werden (Tab. 22).

**Table 22:** 

	Empfehlung	Empfehlungsgrad	
E43	Der präoperative Neubeginn einer Therapie mit β‑Rezeptorantagonisten bei PatientInnen mit > 2 Risikofaktoren für kardiovaskuläre Ereignisse, die sich einer Operation mit hohem eingriffsbezogenen Risiko unterziehen müssen, KANN erwogen werden [5, 6, 10].	0	⇔
E44	Der präoperative Neubeginn einer Therapie mit β‑Rezeptorantagonisten KANN bei PatientInnen mit KHK und Myokardischämie unter Belastung erwogen werden [5, 6, 10].	0	⇔
E45	Der routinemäßige Neubeginn einer Therapie mit β‑Rezeptorantagonisten präoperativ SOLL NICHT durchgeführt werden.	A	⇓⇓
E46	Die präoperative Fortführung der Therapie mit β‑Rezeptorantagonisten SOLL durchgeführt werden.	A	⇑⇑
E47	Die Unterbrechung der Gabe von Diuretika am Operationstag KANN erwogen werden [5, 6, 10].	0	⇔
E48	Die Gabe von ACEI/ARB SOLLTEN am OP-Tag pausiert werden.	B	⇑

*ACEI* Angiotensin Converting Enzyme (ACE)-Hemmer, *ARB* Angiotensin-II-Rezeptorantagonisten

### C.2 Antidiabetika

Zur Behandlung eines Typ-II-Diabetes mellitus werden primär orale Antidiabetika eingesetzt. Hierzu zählen Sulfonylharnstoffe (z. B. Glibenclamid) und Analoga (Glinide, z. B. Repaglinid), Biguanide (Metformin) ebenso wie Glucosidasehemmer (z. B. Acarbose), Glitazone (z. B. Pioglitazon) und Gliptine (z. B. Sitagliptin). Als neuere Substanzen kommen inzwischen Natrium-Glucose-Cotransporter‑2 (SGLT-2) -Inhibitoren (Gliflozine) und Glucagon-like Peptidase-1(GLP-1)-Agonisten (Inkretinmimetika, Glutide) zum Einsatz.

#### C.2.1 Natrium-Glukose-Cotransporter-2-Inhibitoren

SGLT-2-Inhibitoren hemmen Natrium-Glucose-Cotransporter‑2 Kanäle im Nierentubulus und führen damit zu einer gesteigerten Glukoseausscheidung und Senkung der Blutglukosekonzentration. Außerdem wird die Natriurese gesteigert und das Renin-Angiotensin-Aldosteron-System gehemmt. Aufgrund seiner positiven Effekte, auch bei Nichtdiabetes-Patientinnen und -Patienten mit symptomatischer, chronischer Herzinsuffizienz und reduzierter linksventrikulärer Funktion, wird diese Medikamentengruppe voraussichtlich zukünftig häufiger eingesetzt. Ein Vertreter (Dapagliflozin) ist in der EU aufgrund seiner Vorteile bei kardiovaskulären Erkrankungen seit November 2020 auch für Nichtdiabetiker zugelassen.

Ein relevantes Hypoglykämierisiko besteht nicht. Jedoch ist eine relevante substanzspezifische Nebenwirkung die Entstehung einer „euglykämen“ diabetischen Ketoazidose (EDKA) [102]. Risikofaktoren für die Entstehung einer EDKA sind v. a. Zustände mit einer katabolen Stoffwechsellage (Nahrungskarenz, perioperative Ausschüttung von Cortisol etc.), was ein erhöhtes Risiko für hospitalisierte, operative und kritisch kranke Patientinnen und Patienten erklärt. Außerdem kann es zu vermehrtem renalen Flüssigkeitsverlust kommen. Daher sollte die Therapie mit SGLT-2-Inhibitoren mindestens 24 h, besser 48 h vor Operationen mit niedrigem eingriffsbezogenen Risiko pausiert werden. Vor Operationen mit mittlerem und hohem eingriffsbezogenen Risiko, zu erwartenden Volumenverschiebungen oder bei akut eingeschränkten Organfunktionen sollte die Therapie mit SGLT-2-Inhibitoren mindestens 72 h vor dem Eingriff ausgesetzt werden ^**E49**^. Dementsprechend ist eine engmaschige Kontrolle des Blutzuckers für diese Patienten notwendig, um eine Hyperglykämie zu detektieren (Tab. 23).

**Table 23:** 

	Empfehlung	Empfehlungsgrad	
E49	SGLT-2-Inhibitoren SOLLTEN bei Operationen mit niedrigem eingriffsbezogenen Risiko mindestens 24–48 h und Operationen mit mittlerem und hohem eingriffsbezogenen Risiko mindestens 72 h präoperativ pausiert werden.	B	⇑
E50	Sulfonylharnstoffe und -analoga, GLP-1-Analoga und Alpha-Glucosidase-Hemmer SOLLTEN am Tag der Operation pausiert werden. Bei GLP-1-Agonisten, die nur einmal pro Woche verabreicht werden, sollte der letzte Applikationszeitpunkt des Medikamentes eine Woche vor der geplanten Operation liegen.	B	⇑
E51	Metformin SOLLTE bei Operationen mit niedrigem oder mittlerem eingriffsbezogenen Risiko für Gewebshypoxie, Leber- oder Niereninsuffizienz bis zum Vorabend vor der Operation fortgeführt werden.	B	⇑
E52	Metformin SOLL bei Operationen mit hohem eingriffsbezogenen Risiko für Gewebshypoxie, Leber- oder Niereninsuffizienz 48 h präoperativ pausiert werden.	A	⇑⇑

*SGLT‑2* Sodium-Glucose-Cotransporter‑2, *GLP‑1* Glucagon-like Peptidase‑1

#### C.2.2 Glucagon-like Peptide‑1-Agonisten

Glucagon-like Peptide‑1(GLP‑1)-Agonisten sind synthetisch hergestellte Polypeptide, die die Insulinsekretion in den Betazellen steigern und die Freisetzung von Glukagon in den Alphazellen des Pankreas hemmen. Vermittelt über spezifische Rezeptoren im Magen-Darm-Trakt verzögern sie die Magenentleerung und wirken zentral in der Regulation des Appetits und des Sättigungsgefühls [103]. Diese Eigenschaften begründen den Einsatz der GLP‑1-Agonisten zur Gewichtsreduktion [156]. Studien zeigten außerdem eine Reduktion kardiovaskulärer Ereignisse [157] und eine verbesserte Nierenfunktion [104]. Die Therapie mit GLP‑1-Agonisten ist assoziiert mit gastrointestinalen Effekten und Nebenwirkungen. Hierzu zählen Übelkeit, Erbrechen, Durchfall, Bauchschmerzen und die o. g. verzögerte Magenentleerung. In einer Querschnittstudie wurden bei Patientinnen und Patienten, die sich einem operativen Eingriff unterziehen mussten, trotz Einhaltung der präoperativen Nüchternheit Speisereste nachgewiesen [158]. Basierend auf Fallberichten besteht derzeit die Überlegung, dass die ununterbrochene Einnahme von GLP‑1-Agonisten mit einem erhöhten Risiko für eine Regurgitation von Mageninhalt und pulmonale Aspiration im Rahmen einer Allgemeinanästhesie oder einer Analgosedierung assoziiert sein könnte [105]. Vor dem Hintergrund dieser Berichte hat die amerikanische anästhesiologische Fachgesellschaft (American Society of Anesthesiologists, ASA) bereits konkrete Empfehlungen für den präoperativen Umgang mit GLP‑1-Agonisten publiziert [106]. Trotz der bisher spärlichen Datenlage schließen sich die Autorinnen und Autoren dieser Empfehlung an. Bei elektiven Eingriffen sollte die Therapie mit einem täglich eingenommenen GLP‑1-Agonisten am OP-Tag unterbrochen werden. Bei GLP‑1-Agonisten, die nur einmal pro Woche verabreicht werden, sollte der letzte Applikationszeitpunkt des Medikamentes eine Woche vor der geplanten Operation liegen E50. Diese Zeiträume sind unabhängig von der Indikation (Diabetes Mellitus Typ 2, Gewichtsreduktion) mit einem GLP‑1-Agonisten zu beachten. Nach dem Absetzen des Medikamentes ist eine engmaschige Kontrolle des Blutzuckers erforderlich. Gleichzeitig muss berücksichtigt werden, dass aufgrund einer durch das Medikament verursachten verzögerten Magenentleerung das Aspirationsrisiko bei Patientinnen und Patienten unter Therapie mit GLP‑1-Agonisten erhöht sein kann [105]. Bei fehlender Medikamentenpause oder gastrointestinalen Symptomen wie Übelkeit, Erbrechen oder abdominellen Schmerzen sollte die Patientin oder der Patient für einen elektiven Eingriff als nicht nüchtern betrachtet werden. In diesem Fall ist eine individualisierte und interdisziplinäre Risikobewertung für die Durchführung bzw. Verschiebung des Eingriffs zu empfehlen.

#### C.2.3 Metformin

Metformin kann bei Kumulation (z. B. Niereninsuffizienz, Hypoxie und Leberdysfunktion) in seltenen Fällen zu einer lebensbedrohlichen Laktazidose führen, so dass in der Risikoinformation des BfArM ein Absetzen 48 h vor dem Eingriff empfohlen wird.

Bei Vorliegen von Risikofaktoren für eine Laktazidose sollte Metformin rund 48 h und bei geplanter intravenöser Gabe von Kontrastmittel 24–48 h vor einem operativen Eingriff oder einer Intervention pausiert werden [107, 108]. Im direkt perioperativen Bereich scheint das Risiko der Laktazidose jedoch äußerst gering zu sein [109]. Nach individueller Nutzen-Risiko-Abwägung ist daher auch eine Weiterführung der Medikation bis zum Vorabend der Operation zu rechtfertigen. Die britischen „National Health Services“ (NHS) sowie die „Society for Ambulatory Anesthesia“ (SAMBA) empfehlen sogar die Fortführung der Metformintherapie bei Nierengesunden, u. a. wegen Hinweisen auf ein verbessertes Outcome bei diesem Vorgehen [110]. Die Autoren der hier vorliegenden Empfehlungen sprechen sich für eine Fortführung der Therapie mit Metformin bis zum Vorabend der Operation bei Eingriffen mit niedrigem und mittlerem eingriffsbezogenen Risiko für das Auftreten von Gewebshypoxie, Leber- oder Niereninsuffizienz aus ^**E51**^. Allerdings soll Metformin bei Operationen mit hohem eingriffsbezogenen Risiko für Gewebshypoxie, Leber- oder Niereninsuffizienz 48 h vor dem Eingriff pausiert werden (z. B. große abdominalchirurgische Eingriffe, große orthopädische Eingriffe) ^**E52**^ ([111]; Tab. 23).

#### C.2.4 Sulfonylharnstoffe

Sulfonylharnstoffe beeinflussen die Pathophysiologie des Diabetes mellitus durch eine Steigerung der Insulinsekretion und Proliferation der Beta-Zellen des Pankreas [112]. Sulfonylharnstoffe und -analoga sollten aus diesem Grund am Tag der Operation, bei Nüchternphasen oder akut eingeschränkten Organfunktionen auch noch früher, pausiert werden [113] ^**E50**^.

Sulfonylharnstoffe verhindern im Tierexperiment die durch Ischämie bzw. volatile Anästhetika induzierte, insbesondere myokardiale Präkonditionierung und vergrößern so das Nekroseareal des Myokards nach Ischämie. Ob ähnliche Effekte auch beim Menschen auftreten und Sulfonylharnstoffe daher präoperativ abgesetzt werden sollten, ist bislang jedoch unklar [114].

#### C.2.5 Thiazolidindione, ‚Insulinsensitizer‘

Glitazone erhöhen die Sensitivität verschiedener Gewebe gegenüber Insulin und werden zunehmend bei Patientinnen und Patienten mit Diabetes mellitus Typ II zur Glucosekontrolle eingesetzt [115]. Nach derzeitigen Erkenntnissen scheint eine Pausierung bei Einnahme als Monotherapie nicht notwendig zu sein.

#### C.2.6 DPP4-Inhibitoren

Die Wirkung von DPP4-Inhibitoren (Gliptinen) entspricht der Wirkung des körpereigenen Hormons Inkretin und senkt über eine vermehrte Insulinfreisetzung aus den β‑Zellen und eine erhöhte Glukagonsynthese in den α‑Zellen des Pankreas den Blutzucker [103]. Hypoglykämien treten bei einer Monotherapie mit Gliptinen in der Regel nicht auf. Auch diese Medikamente müssen präoperativ nicht pausiert werden, wenn sie zur Monotherapie des Diabetes mellitus eingesetzt werden.

#### C.2.7 Insuline

Insuline stellen die Standardtherapie bei Diabetes Typ I sowie bei fortgeschrittenem Diabetes Typ II mit Wirkungslosigkeit oraler Antidiabetika dar. Bei der Insulintherapie wird eine konventionelle Insulintherapie von einer intensivierten Insulintherapie (mit einer Kombination aus einem langwirkendem „Basalinsulin“ und einem kurzwirksamen Normalinsulin zu den Mahlzeiten) und einer Insulinpumpentherapie unterschieden. Durch die präoperative Nüchternheit und des je nach Art und Größe des Eingriffes bestehenden Postaggressionsstoffwechsels ergibt sich bei fortdauernder Insulintherapie zum einen das Risiko einer Hypoglykämie. Zum anderen steigt jedoch im Falle einer Dosisreduktion die Gefahr einer Ketoazidose aufgrund der relativen Insulinresistenz im Postaggressionsstoffwechsel.

Bei kurzdauernden Eingriffen und präoperativ bestehender, intensivierter Insulintherapie sollte am Morgen des OP-Tages lediglich die Basisinsulintherapie ohne zusätzliche Bolusgabe erfolgen. Postoperativ kann dabei die übliche Insulintherapie fortgesetzt werden [116]. Engmaschige Kontrollen und gegebenfalls Korrekturen des Blutzuckers sind während des gesamten perioperativen Verlaufs unerlässlich. Eine Dosisreduktion von Langzeitinsulinen am Vorabend bzw. am Tag der Operation um maximal 50 % kann erwogen werden, wenn

- eine deutliche Dosisanpassung vor kurzer Zeit erfolgt ist,
- der BZ der Patientin bzw. des Patienten unzureichend eingestellt ist, d. h. häufig niedrige oder zu hohe (nüchtern) BZ-Werte auftreten,
- die geplante Operation einen erwartbaren, deutlichen Einfluss auf die Glukosehomöostase hat (OP-Dauer über 2 h, relevanter Blutverlust, relevante Volumenverschiebung etc.),
- eine (akute) Nieren- oder Leberinsuffizienz besteht.

Eine Übersicht über die Empfehlungen zum perioperativen Umgang mit oralen Antidiabetika gibt Tab. 24.

**Table 24:** 

Medikamentengruppe	Risiken (Auswahl)	Medikamentenpause
Sulfonylharnstoffe und -analoga, ‚Glinide‘	Höchstes Hypoglykämie-Risiko, besonders bei geriatrischen PatientInnen, Polypharmazie, längeren Nüchternzeiten und Organdysfunktionen	Am Operationstag
Biguanide „Metformin“	Laktatazidose	Am Operationstag: bei Operationen mit niedrigem oder mittlerem eingriffsbezogenen Risiko für Gewebshypoxie, Leber- oder Niereninsuffizienz. 48 h präoperativ bei Operationen mit hohem eingriffsbezogenen Risiko für Gewebshypoxie, Leber- oder Niereninsuffizienz
SGLT-2-Inhibitoren „Gliflozine“	Euglykämische Diabetische Ketoazidose (EDKA)	24 bis > 72 h präoperativ in Abhängigkeit vom Zustand der Patientin bzw. des Patienten und Operation
GLP-1-Analoga „Glutide“	Verzögerte Magenentleerung, Übelkeit, Erbrechen	Am Operationstag bei täglicher Einnahme. Eine Woche vor dem Operationstag bei wöchentlicher Einnahme
DPP4-Inhibitoren „Gliptine“	–	Keine Pause
Alpha-Glucosidase-Hemmer „Acarbose“	–	Am Operationstag
Thiazolidindione ‚Insulinsensitizer‘ ‚Glitazone‘	Ödeme, kardiale Dekompensation	Keine Pause

### C.3 HMG-CoA-Reduktase-Inhibitoren (Statine)

Die als Lipidsenker eingesetzten HMG-CoA-Reduktase-Inhibitoren (Statine) stabilisieren vulnerable Plaques, wirken schnell anti-inflammatorisch und anti-thrombotisch und können die Inzidenz perioperativer Ischämien, von (Re‑)Infarkten und Todesfällen bei Patientinnen und Patienten mit koronarem Risiko senken [119]. Bei Patientinnen und Patienten, bei denen eine Statintherapie besteht, soll diese Therapie perioperativ fortgesetzt werden [5, 6, 120] ^**E53**^. Bei Patientinnen und Patienten, bei denen, unabhängig vom operativen Eingriff, eine Indikation für eine Statintherapie besteht, sollte bereits präoperativ mit der Statinbehandlung begonnen werden ^**E54**^. Darüber hinaus kann eine präoperative hochdosierte Gabe von schnellwirksamen Statinen (reload therapy) bei Patientinnen und Patienten mit einer koronaren Herzerkrankung erwogen werden ([121–123]; Tab. 25).

**Table 25:** 

	Empfehlung	Empfehlungsgrad	
E53	Bei PatientInnen, bei denen eine Statintherapie besteht, SOLL diese Therapie perioperativ fortgesetzt werden [5, 6, 10].	A	⇑⇑
E54	Bei PatientInnen, bei denen, unabhängig vom operativen Eingriff, eine Indikation für eine Statintherapie besteht, SOLLTE bereits präoperativ der Beginn einer Statintherapie erwogen werden.	B	⇑

### C.4 Antikoagulantien

Bei der Planung operativer Eingriffe von antikoagulierten Patientinnen und Patienten müssen individuelle sowie Prozeduren-relevante Risiken (Tab. 26) für Blutung und Thromboseentstehung berücksichtigt werden. In vorangegangenen Studien konnte gezeigt werden, dass das perioperative antithrombotische Management erheblichen Einfluss auf Blutungskomplikationen [124] und Letalität [125] hat. Für eine detaillierte Betrachtung der Antikoagulation bei Regionalanästhesieverfahren verweisen wir auf die aktuelle S1-Leitlinie der DGAI zu „Rückenmarknahe Regionalanästhesien und Thromboembolieprophylaxe/antithrombotische Medikation“ [126]. Das eingriffsspezifische Blutungsrisiko ist in Tab. 26 gezeigt und soll zur präoperativen Risikoeinschätzung herangezogen werden [5, 6, 10]. Umstellungen der medikamentösen antithrombotischen Therapie sollen präoperativ mit dem Patienten besprochen werden.

**Table 26:** 

Niedriges Blutungsrisiko	Mittleres Blutungsrisiko	Hohes Blutungsrisiko
Katarakt oder Glaukom	Abdominalchirurgie	Abdominalchirurgie mit Leberbiopsien
Zahneingriffe	Mammachirurgie	Ausgedehnte Tumorchirurgie
Endoskopie ohne Biopsie oder Resektion	Orthopädische Chirurgie der Extremitäten	Neurochirurgie
Oberflächliche Chirurgie	Komplexe zahnchirurgische Eingriffe	Rekonstruktive plastische Chirurgie
	Endoskopie mit einfacher Biopsie	Thoraxchirurgie
	Gastroskopie oder Koloskopie mit einfacher Biopsie	Urologische Chirurgie
		Große orthopädische Chirurgie
		Spezifische Interventionen

#### C.4.1 Orale Antikoagulantien

Das perioperative Management oraler Antikoagulantien schließt die Therapie mit spezifischen Vitamin-K-Antagonisten oder Direkte Orale Antikoagulantien (DOAK) ein. Bei der präoperativen Evaluation und der Gesamteinschätzung des Risikos sind neben der Medikamentenanamnese insbesondere das eingriffsspezifische Blutungsrisiko sowie patientenspezifische Risikofaktoren zu berücksichtigen.

##### C.4.1.1 Vitamin-K-Antagonisten.

Zurzeit stehen in Deutschland 2 Vitamin-K-Antagonisten zur Verfügung: Warfarin (Halbwertszeit 36–48 h) und Phenprocoumon (Halbwertszeit 100 h). Patientinnen und Patienten mit mechanischen Herzklappen sind immer und Patientinnen und Patienten mit biologischen Herzklappen meist noch in den ersten 3 Monaten nach Implantation mit Vitamin-K-Antagonisten antikoaguliert. Je nach Klappentyp und -lokalisation wird eine INR von 2,0–3,5 angestrebt. Weitere wichtige klinische Indikationen für die Gabe von Vitamin-K-Antagonisten sind die postthrombotische Therapie sowie das Vorhofflimmern. Das Risiko für thrombembolische Komplikationen bei perioperativer Unterbrechung der Antikoagulation muss in jedem Einzelfall individualisiert gegen das perioperative Blutungsrisiko abgewogen werden. Im Falle eines Absetzens erfolgt der Therapiestopp 3–5 Tage (bei Phenprocoumon besser 5–8 Tage) präoperativ unter täglicher Kontrolle des INR (Ziel für Operation: < 1,5) (Abb. 3).

##### C.4.1.2 Vitamin-K-Antagonisten bei Patientinnen und Patienten mit mechanischem Herzklappenersatz.

Für Patientinnen und Patienten mit mechanischen Herzklappen ist der therapeutische INR-Wert entscheidend zur Prävention thrombembolischer Komplikationen. Bei kleinen chirurgischen Eingriffen und Eingriffen, bei denen die Blutung sehr gut zu kontrollieren ist, kann die Gabe von Vitamin-K-Antagonisten fortgeführt werden ^**E55**^. Bei großen chirurgischen Eingriffen, die einen INR < 1,5 erfordern, soll die Applikation von Vitamin-K-Antagonisten unterbrochen werden ^**E56**^ [5, 6, 10]. Im PERI-OP Trial wurde die Überbrückung mit dem niedermolekularen Heparin (NMH) Dalteparin bei Patientinnen und Patienten mit mechanischem Herzklappenersatz oder Vorhofflimmern und chirurgischen Eingriffen untersucht. Die Inzidenz thrombembolischer Komplikationen konnte durch Dalteparin nicht gesenkt werden und die Inzidenz für schwere postoperative Blutungen veränderte sich durch Therapie mit dem niedermolekularen Heparin nicht [127]. In anderen Untersuchungen konnte gezeigt werden, dass die Überbrückungstherapie mit dem NMH Dalteparin mit einer deutlichen Zunahme an schweren perioperativen Blutungen einhergeht [5, 6, 10, 127, 128]. Bei Patientinnen und Patienten mit mechanischem Herzklappenersatz und hohem eingriffsspezifischen Risiko für thrombembolische Komplikationen sollte die perioperative Unterbrechung der Therapie mit Vitamin-K-Antagonisten erwogen werden. Gleichzeitig muss eine Behandlung („Bridging“) mit niedermolekularem Heparin in Betracht gezogen werden [5, 6, 10, 129].

##### C.4.1.3 Vitamin-K-Antagonisten bei Patientinnen und Patienten mit Vorhofflimmern oder venösen Thrombosen.

Bei Patientinnen und Patienten mit Vorhofflimmern oder venösen Thrombosen kann die Gabe von Vitamin-K-Antagonisten bei elektiven, nicht herz-thoraxchirurgischen Operationen mit niedrigem und mittlerem eingriffsbezogenen Blutungsrisiko fortgeführt werden ^**E55**^. Bei Operationen mit hohem eingriffsbezogenen Blutungsrisiko stellt sich in der „BRIDGE“ Untersuchung [128] eine 3–5 tägige Pause mit Warfarin ohne Bridging verglichen mit der Heparin-Gabe als gleichwertig hinsichtlich dem Auftreten thrombembolischer Komplikationen dar. Gleichzeitig zeigte sich in der Gruppe, die nach dem Absetzen von Warfarin ohne Heparin behandelt wurde, eine signifikant geringere Inzidenz für perioperative Blutungen. In Übereinstimmung hierzu fand sich in einer prospektiven Registerstudie eine erhöhte Blutungsrate sowie eine erhöhte Letalität bei Patientinnen und Patienten, die nach dem Absetzen von Vitamin-K-Antagonisten mit niedermolekularem Heparin behandelt wurden [130]. Die Indikation für eine Therapie mit niedermolekularem Heparin nach Absetzen eines Vitamin-K-Antagonisten ist daher zunehmend kritisch zu stellen. Bei Patientinnen und Patienten mit hohem Thrombembolierisiko (Vorhofflimmern mit einem CHA_2_DS_2_-VASc Score > 6, Thrombophilie, thrombembolischer Schlaganfall < 3 Monate) wird ein Therapieersatz mit niedermolekularem Heparin nach wie vor meist als indiziert angesehen, wenn sich der INR im subtherapeutischen Bereich befindet [5, 6]. Die letzte Gabe von niedermolekularem Heparin sollte mindestens 12 h (bei Störungen der Nierenfunktion entsprechend länger) vor einem operativen Eingriff erfolgt sein. Die Entscheidung zur perioperativen venösen Thromboembolieprophylaxe durch unfraktioniertes oder niedermolekulares Heparin bleibt von diesen Überlegungen unberührt [131].

#### C.4.2 Direkte Orale Antikoagulantien (DOAK)

DOAK sind eine verfügbare Klasse von oral/enteral applizierbaren Faktor IIa- oder Xa-Antagonisten. Derzeit sind 4 Medikamente verfügbar: Dabigatran (Faktor IIa Inhibitor) sowie Apixaban, Rivaroxaban und Edoxaban (Fxa Inhibitoren). Die derzeitigen Indikationen sind die Prophylaxe von Schlaganfällen und systemischen Embolien bei nichtvalvulärem Vorhofflimmern, die Therapie und Sekundär-Prophylaxe der tiefen Venenthrombose und der Lungenembolie sowie die Thromboembolie-Prophylaxe bei elektiver Hüft- und Kniegelenk-Chirurgie.

DOAK (HWZ 9–14 h) werden mit Ausnahme von Dabigatran (überwiegend renal) auf verschiedenen Wegen eliminiert. Bei Operationen mit niedrigem eingriffsbezogenen Blutungsrisiko kann die Therapie mit DOAK präoperativ fortgesetzt und der Eingriff 12 oder 24 h nach der letzten Einnahme durchgeführt werden ^**E57**^. Bei Eingriffen mit mittlerem eingriffsbezogenen Blutungsrisiko wird in Abhängigkeit von der GFR der Niere die Therapie mit DOAK > 24 h ^**E58**^ vorher abgesetzt. Bei Operationen mit hohem eingriffsbezogenen Blutungsrisiko sollte das DOAK wiederum in Abhängigkeit von der GFR der Niere > 48 h vor dem operativen Eingriff abgesetzt werden (Tab. 27; [5, 6, 10, 132]).

**Table 27:** 

**Niedriges Blutungsrisiko**				
Die Einnahme wird nicht unterbrochen. Der Eingriff soll 12 oder 24 h nach der letzten Einnahme durchgeführt werden (in Abhängigkeit von der einmaligen oder zweimaligen Gabe des Medikaments)				
	**Mittleres Blutungsrisiko**	**Hohes Blutungsrisiko**	**Mittleres Blutungsrisiko**	**Hohes Blutungsrisiko**
*Renale Funktion (GFR, ml/min)*	Dabigatran	Dabigatran	Apixaban, Rivaroxaban, Edoxaban	Apixaban, Rivaroxaban, Edoxaban
*>* *80*	> 24 h	> 48 h	> 24 h	> 48 h
*50–79*	> 36 h	> 72 h	> 24 h	> 48 h
*30–49*	> 48 h	> 96 h	> 24 h	> 48 h
*15–29*	Nicht indiziert	Nicht indiziert	> 36 h	> 48 h

*DOAK* Direkte Orale Antikoagulantien

Ob ein DOAK präoperativ belassen, gemäß Abklingzeit mit ausreichendem Abstand zur Operation abgesetzt (Cave: zu frühes Absetzen vermeiden!) oder aber auf Heparin umgestellt werden sollte, hängt von der Art der Operation und deren Blutungsrisiko sowie von der ursprünglichen Indikation für die Antikoagulation ab. In Studien konnte gezeigt werden, dass die Ersatztherapie mit Heparin oder niedermolekularem Heparin mit einem höheren Blutungsrisiko einhergeht, jedoch zu keiner Reduktion thrombembolischer Komplikationen führt [133–135]. Die präoperative Ersatztherapie mit Heparin ist deshalb generell nicht empfohlen [5, 6] und sollte individualisiert nur in ausgewiesenen Fällen in Erwägung gezogen werden, u. a. bei Patientinnen und Patienten mit einem thrombembolischen Ereignis in den zurückliegenden 3 Monaten oder die bei einem vorausgegangenen Absetzen des DOAK ein thrombembolisches Ereignis entwickelt haben [5, 6, 10]. Die Entscheidung hierüber sollte in enger Absprache zwischen den beteiligten Fachdisziplinen (i. d. R. Chirurgie, Anästhesie und Innere Medizin) fallen.

#### C.4.3 Einfache Thrombozytenaggregationshemmung

Bei Patientinnen und Patienten mit bekannten kardiovaskulären Vorerkrankungen gehört niedrig-dosierte Acetylsalicylsäure (ASS) in der Prävention neuer kardiovaskulärer Ereignisse zur empfohlenen Dauermedikation [136]. Die POISE‑2 Studie untersuchte den Einfluss der perioperativen Gabe von ASS bei Patientinnen und Patienten, die sich einem elektiven, nicht herz-thoraxchirurgischen Eingriff unterziehen mussten [137]. Bei 10.010 Patienten konnte gezeigt werden, dass niedrig-dosiertes ASS bis zum 30. postoperativen Tag zu keiner Reduktion neu aufgetretener Myokardinfarkte oder einer Abnahme der Letalität führt. Allerdings waren Blutungskomplikationen in der Gruppe mit niedrig-dosiertem ASS häufiger feststellbar. In einer Subgruppen-Analyse an 470 Patienten, die sich zu unterschiedlichen Zeitpunkten vor dem operativen Eingriff einer perkutanen Koronarintervention unterziehen mussten, konnte jedoch eine signifikante Reduktion der Letalität oder neu aufgetretener Herzinfarkte nachgewiesen werden. Aus den genannten Gründen wird deshalb empfohlen, dass bei Patientinnen und Patienten, die kein erhöhtes eingriffsbezogenes Blutungsrisiko haben und sich vor der Operation einer perkutanen Koronarintervention unterziehen mussten, die Gabe von ASS perioperativ weiter fortzuführen ^**E59**^ [5, 6]. ASS sollte bei Patientinnen und Patienten, die sich einer Operation mit hohem eingriffsbezogenen Blutungsrisiko (s. Tab. 26) unterziehen müssen, 7 Tage vor dem operativen Eingriff abgesetzt werden [5, 6]. Bei Patientinnen und Patienten, die sich nach einer Transkatheter-Aortenklappen-Intervention (TAVI) einem nicht kardiochirurgischen Eingriff unterziehen müssen, gibt es aktuell keine Empfehlung zur Fortsetzung oder dem Absetzen der niedrig dosierten ASS-Therapie [138].

Patientinnen und Patienten, die mit einer P2Y_12_-Inhibitor Monotherapie behandelt werden, benötigen eine individualisierte Abwägung hinsichtlich des perioperativen Blutungs- vs. Ischämie-Risikos. Es gibt keine eindeutigen Empfehlungen in der Literatur, die eine Fortführung der P2Y_12_-Inhibitior Monotherapie, den Wechsel zu ASS, eine kurze Unterbrechung der Therapie mit P2Y_12_-Inhibitoren oder ein Bridging-Verfahren für einen chirurgischen Eingriff favorisieren [5, 6].

##### C.4.3.1 Duale Thrombozytenaggregationshemmung.

In Observationsstudien konnte bei Patientinnen und Patienten mit akutem Koronarsyndrom (ACS) und perkutaner koronarer Intervention gezeigt werden, dass die Inzidenz für MACE, einschließlich Tod, Herzinfarkt oder Stent-Thrombose, bei nicht kardiochirurgischen Eingriffen um 2–8 % erhöht ist [139, 140]. Es besteht die Indikation für eine duale Thrombozytenaggregationshemmung, die die Kombination aus ASS und einen P2Y_12_-Antagonisten (Clopidogrel bzw. Ticagrelor/Prasugrel) vorsieht. Der Nutzen dieser dualen Thrombozytenaggregationshemmung nach operativer bzw. interventioneller koronarer Revaskularisierung (z. B. Stent), aber auch bei Patientinnen und Patienten mit stattgehabtem akutem Koronarsyndrom ist klar belegt [141].

Es wird empfohlen, bei Patientinnen und Patienten innerhalb der ersten 6 Monate nach perkutaner koronarer Intervention oder innerhalb von 12 Monaten nach akutem Koronarsyndrom und medikamentöser Therapie mit dualer Thrombozytenaggregationshemmung keine elektiven operativen Eingriffe durchzuführen ^**E60**^ [5, 6, 10, 80, 142]. Neue Daten zeigen, dass die Implantation moderner Koronarstents, in Kombination mit einer dualen Thrombozytenaggregationshemmung für 1–3 Monate, das Risiko für MACE und Stentthrombosen deutlich reduziert. Aus diesem Grund kann bei Patientinnen und Patienten eine zeitkritische elektive Operation mit niedrigem oder mittlerem eingriffsbezogenen Risiko für kardiovaskuläre Ereignisse frühestens 1 Monat und bei hohem eingriffsspezifischen Risiko frühestens 3 Monate nach Beginn der Therapie mit Thrombozytenaggregationshemmern erwogen werden [5, 6, 10].

Bei zeitkritischen, elektiven operativen Eingriffen wird folgender präoperativer Umgang mit der dualen Thrombozytenaggregationshemmung empfohlen: In Erwägung gezogen werden muss der Wechsel von hoch potenten P2Y_12_-Inhibitoren Prasugrel oder Ticagrelor auf Clopidogrel oder die Beendigung der ASS-Therapie unter Verwendung einer Prasugrel- oder Ticagrelor-Monotherapie [5, 6, 10]. Falls ein völliger Verzicht von P2Y_12_-Antagonisten für den operativen Eingriff erforderlich ist, werden nach derzeitigem Kenntnisstand die P2Y_12_-Antagonisten Ticagrelor für 3–5 Tage, Clopidogrel für 5 Tage und Prasugrel für 7 Tage vor Operationen mit hohem eingriffsbezogenen Blutungsrisiko abgesetzt. In dieser Zeit wird die Therapie mit ASS fortgeführt. Das Risiko für starke Blutungen reduziert sich hierdurch um 50 %, ohne das Risiko für MACE oder Tod zu erhöhen ^**E61**^ [143]. Bei hohem Risiko für ein myokardiales ischämisches Ereignis nach Stentimplantation muss diese Entscheidung individuell und interdisziplinär abgestimmt werden. Zwingend ist ein Absetzen vor Eingriffen in geschlossenen Höhlen (Augenhinterkammer, intraspinale und intrazerebrale Eingriffe) sowie vor rückenmarksnaher Regionalanästhesie. Auch die perioperative Therapie mit ASS muss individuell erfolgen. Bei Patientinnen und Patienten mit hohem kardiovaskulären Risiko (rezidivierende Angina pectoris, Zustand nach akutem Koronarsyndrom, Zustand nach Koronarintervention mit Stentimplantation) sollte die niedrigdosierte Gabe von ASS nur bei absoluter Kontraindikation perioperativ beendet werden (Übersicht bei [141]; Abb. 4; Tab. 28).

**Table 28:** 

	Empfehlung	Empfehlungsgrad	
E55	Bei kleinen und mittleren chirurgischen Eingriffen KANN die Gabe von Vitamin-K-Antagonisten fortgeführt werden [5, 6, 10].	0	⇔
E56	Bei großen chirurgischen Eingriffen SOLL die Applikation von Vitamin-K-Antagonisten unterbrochen werden [5, 6, 10].	A	⇑⇑
E57	Operationen mit niedrigem eingriffsbezogenen Blutungsrisiko SOLLEN 12–24 h nach letzter Einnahme eines DOAK durchgeführt werden [5, 6, 10].	A	⇑⇑
E58	Die Gabe von DOAK SOLL bei Operationen mit mittlerem eingriffsbezogenen Blutungsrisiko in Abhängigkeit von der Nierenfunktion für > 24–48 h unterbrochen werden [5, 6, 10].	A	⇑⇑
E59	Die Gabe von DOAK SOLL bei Operationen mit hohem eingriffsbezogenen Blutungsrisiko in Abhängigkeit von der Nierenfunktion für > 48–96 h unterbrochen werden [5, 6, 10].	A	⇑⇑
E60	Die perioperative Gabe von ASS SOLL fortgesetzt werden, wenn das Blutungsrisiko kalkulierbar ist.	A	⇑⇑
E61	Bei Operationen mit hohem eingriffsbezogenen Blutungsrisiko SOLL die Gabe von ASS 7 Tage präoperativ abgesetzt werden [5, 6, 10].	A	⇑⇑
E62	Elektive, nicht herz-thoraxchirurgische Operationen SOLLEN um 6 Monate nach elektiver Koronarintervention und 12 Monate nach akutem Koronarsyndrom und medikamentöser Therapie mit dualer Thrombozytenaggregationshemmung verschoben werden [5, 6, 10].	A	⇑⇑
E63	Die Unterbrechung der Therapie mit P2Y_12_-Inhibitoren SOLL vor elektiven operativen Eingriffen bei Ticagrelor für 3–5 Tage, Clopidogrel für 5 Tage und Prasugrel für 7 Tage erfolgen [5, 6, 10].	A	⇑⇑

*DOAK* Direkte Orale Antikoagulantien

#### C.4.4 Psychopharmaka

Die Modifikation der psychiatrischen Dauermedikation ist im Rahmen der präoperativen Risikoevaluation ein wichtiger Aspekt. Grundsätzlich gilt, dass eine bestehende Dauermedikation zur Behandlung von Psychosen oder neurologischen Erkrankungen perioperativ fortgeführt werden sollte ^**E64**^. Allerdings bestehen bei einigen Psychopharmaka Neben- und Wechselwirkungen, die perioperativ zu beachten sind. Muss die psychiatrische Pharmakotherapie präoperativ abgesetzt werden, kann es zu einer Reihe von Entzugssyndromen kommen, die es zu beachten gilt (Tab. 29).

##### C.4.4.1 Trizyklische Antidepressiva.

Trizyklische Antidepressiva werden zur Behandlung von Depressionen und in der multimodalen Schmerztherapie eingesetzt. Sie hemmen die Wiederaufnahme von Dopamin, Noradrenalin und Serotonin im Zentralnervensystem (ZNS) und im peripheren Gewebe. Vielfältige Nebenwirkungen können durch die teilweise hemmenden Effekte an einer Vielzahl anderer Rezeptoren (z. B. Histamin‑, Serotonin‑, Noradrenalin-Rezeptoren) entstehen [144, 145]. Berücksichtigt werden muss, dass trizyklische Antidepressiva die QT-Zeit verlängern können. Gleichzeitig kann die Wirkung direkter Sympathomimetika erhöht und die von indirekten abgeschwächt werden. Dies muss besonders bei der Verwendung von Lokalanästhetika mit Adrenalinzusatz berücksichtigt werden. Die Wirkung von Hypnotika, Opioiden und Inhalationsanästhetika kann verstärkt werden.

Trizyklische Antidepressiva werden über das CYP450-System (Cytochrom P450) metabolisiert. Eine Interaktion mit anderen Substanzen, die ebenfalls über dieses Enzymsystem metabolisiert werden, kann nicht ausgeschlossen werden [146]. Eine Dauermedikation sollte perioperativ fortgeführt werden.

##### C.4.4.2 Serotonin-Wiederaufnahmehemmer und Serotonin-Noradrenalin-Wiederaufnahmehemmer.

Serotonin-Wiederaufnahmehemmer (SSRI) und Serotonin-Noradrenalin-Wiederaufnahmehemmer (SNRI) inhibieren die Wiederaufnahme von Serotonin und/oder Noradrenalin im präsynaptischen Spalt. Die abrupte Beendigung der Therapie kann zu Entzugserscheinungen führen. Die gleichzeitige Gabe anderer serotomimetisch wirksamer Substanzen (z. B. Pethidin, Pentazocin, Tramadol, Tapentadol, MAO-Hemmer) können zum Serotonin-Syndrom mit gravierenden Folgen (Hyperthermie, vegetative Instabilität und Bewusstseinsstörungen bis zum Koma) führen [146]. Eine Dauermedikation sollte perioperativ fortgeführt werden.

##### C.4.4.3 Monoaminooxidase-Hemmer.

Monoaminooxidase (MAO)-Hemmer, die in die beiden Subtypen MAO‑A und MAO‑B unterteilt werden, erhöhen ebenfalls die Konzentration von Monoaminen im synaptischen Spalt. Je nach Wirkstoff erfolgt eine selektive oder nichtselektive und reversible oder nicht-reversible Hemmung. Substanzen der 1. Generation (z. B. Tranylcypromin) wirken nicht selektiv und irreversibel auf MAO‑A und MAO‑B. Substanzen der 2. Generation wirken selektiv und irreversibel (Clorgylin auf MAO‑A und Deprenyl auf MAO-B) und Substanzen der 3. Generation selektiv und reversibel (z. B. Moclobemid auf MAO-A). Unter der Therapie mit MAO-Hemmern kommt es zu zahlreichen pharmakologischen Interaktionen: Indirekt wirkende Sympathomimetika können schwer beherrschbare hypertensive Krisen durch die Freisetzung von Noradrenalin hervorrufen. Die Applikation von Pethidin und Tramadol kann zu einem Typ-I-Serotonin-Syndrom führen [147]. Die CYP450-Inhibition kann eine verlängerte Wirkung von Opioiden zur Folge haben. Die aktuelle Studienlage hinsichtlich der präoperativen Nutzung von MAO-Inhibitoren ist nicht eindeutig und vereinzelt wird das Absetzen als nicht mehr zwingend erforderlich gesehen [148]. In anderen Publikationen wird empfohlen die Gabe von reversiblen MAO-Inhibitoren am Vortag des Eingriffs zu pausieren um eine Anästhesie sicher durchführen zu können. Irreversible MAO-Hemmer können im Rahmen elektiver Eingriffe zwei Wochen vor dem Eingriff auf reversible MAO-Hemmer umgestellt werden [143] ^**E65**^.

##### C.4.4.4 Lithium.

Lithium wird in erster Linie zur Behandlung von bipolaren und affektiven Störungen verwendet. Lithium beeinflusst die GABAerge und serotonerge Neurotransmission und kann zu einer hypothyreoten Stoffwechsellage führen. Da Lithium renal eliminiert wird, muss perioperativ eine bestehende oder drohende Verschlechterung der Nierenfunktion beachtet werden [146]. Lithium kann die neuromuskuläre Blockade verlängern, den Anästhetikabedarf verringern und zu einer gesteigerten kardialen Erregungsbildung und -weiterleitung führen. Bei erhöhtem Risiko für eine Lithium-Retention mit Intoxikation und akutem Nierenversagen wird empfohlen, das Medikament 72 h vor einem operativen Eingriff abzusetzen ^**E66**^. Entzugserscheinungen durch abruptes Absetzen des Medikamentes sind nicht zu erwarten [146] (Tab. 29).

##### C.4.4.5 Neuroleptika.

Neuroleptika sind eine sehr heterogene Gruppe von Psychopharmaka mit sedierenden und antipsychotischen Eigenschaften. Sie werden vor allem zur Therapie von Wahnvorstellungen und Halluzinationen bei Schizophrenie und bipolaren Störungen eingesetzt [146]. Aufgrund des Risikos einer Rückkehr psychotischer Symptome ist ein perioperatives Absetzen nicht gerechtfertigt. Berücksichtigt werden müssen die potenziell sedierende Wirkung, eine mögliche QT-Zeit-Verlängerung sowie ein α1-adrenerg-antagonistischer Effekt. Eine Dauermedikation mit Neuroleptika soll perioperativ fortgeführt werden.

##### C.4.4.6 Antikonvulsiva.

Das primäre Therapieziel bei der Epilepsie ist die Anfallsfreiheit oder die Reduktion der Anfallsfrequenz auf ein tolerables Maß. Die präoperative Risikoevaluation schließt die Anfallskontrolle und -frequenz sowie die genaue Analyse der den Patientinnen und Patienten bekannten Triggerfaktoren für die Epilepsie mit ein. Zur Kontrolle der Epilepsie kommen Antikonvulsiva zum Einsatz, die eine Vielzahl anästhesierelevanter Nebenwirkungen aufweisen, u. a. Blutbildveränderungen, Gerinnungsstörungen, Leberfunktionsstörungen, Erregungsleitungsstörungen, allergische Reaktionen, Übelkeit und Erbrechen. Durch die Interaktion der Antikonvulsiva mit Cytochrom-P450-Enzymen der Leber kann der Bedarf an Muskelrelaxanzien und Hypnotika nach längerer Einnahme erhöht sein. In der präoperativen Evaluation kann die Bestimmung der exakten Spiegel der Antikonvulsiva im Blut erforderlich sein. Die antikonvulsive Dauermedikation soll im therapeutischen Bereich perioperativ fortgeführt werden ^**E67**^. Antikonvulsiva werden auch regelmäßig zur Behandlung neuropathischer Schmerzen genutzt. Pregabalin ist ein zugelassenes Medikament bei Angststörungen. Auch für diese Indikation sollte die Dauermedikation perioperativ fortgeführt werden. Gesonderte Aufmerksamkeit erfordert die Gabe von Antikonvulsiva bei neurochirurgischen Eingriffen an Epilepsie- und Hirntumorpatienten (Tab. 29).

**Table 29:** 

	Empfehlung	Empfehlungsgrad	
E64	Eine Dauermedikation mit Psychopharmaka SOLLTE fortgeführt werden.	B	⇑
E65	Ein Austausch von irreversiblen MAO-Inhibitoren durch reversible MAO-Inhibitoren KANN präoperativ erfolgen.	0	⇔
E66	Eine Dauermedikation mit Lithium SOLLTE 72 h vor Operationen pausiert werden.	B	⇑
E67	Die antikonvulsive Dauermedikation soll im therapeutischen Bereich vor elektiven Eingriffen fortgeführt werden.	A	⇑⇑

*MAO* Monoaminooxidase

### C.5 Anti-Parkinson Medikamente

Bei Patienten mit Morbus Parkinson werden häufig Levodopa (L-Dopa), Dopaminagonisten, MAO-B-Hemmer, Catechol-O-Methyltransferase (COMT)-Inhibitoren N‑Methyl-D-Aspartat (NMDA)-Antagonisten und Anticholinergika eingesetzt.

Diese Medikamente erhöhen die Konzentration bzw. Wirkung von Dopamin im Gehirn und wirken durch eine direkte (L-Dopa) oder indirekte (Bromocriptin) Erhöhung von Dopamin oder durch Hemmung des Abbaus (z. B. Selegilin). Das Pausieren oder Absetzen der Medikation kann zu Muskelrigidität bis hin zu schweren Parkinsonkrisen mit Schluck- und Atemproblemen führen [149].

Die Dauermedikation bei Morbus Parkinson soll auch am OP-Tag fortgeführt werden ^**E68**^. Bei unsicherem Schluckakt sollte die intraoperative Anlage einer Magensonde erwogen und die Indikation für eine erweiterte postoperative Überwachung berücksichtigt werden [150, 151].

Dopamin-Antagonisten (z. B. Metoclopramid) und Medikamente mit extrapyramidal-motorischem Nebenwirkungsprofil (z. B. Droperidol, HT3-Antagonisten) sollen bei diesen Patientinnen und Patienten vermieden werden (Tab. 30).

**Table 30:** 

	Empfehlung	Empfehlungsgrad	
E68	Die Dauermedikation bei Morbus Parkinson SOLL auch am OP-Tag fortgeführt werden.	A	⇑⇑

### C.6 Kortikosteroide

Patientinnen und Patienten, die sich einer Kortikosteroid-Dauermedikation (> 5 Tage) unterziehen müssen, unterliegen einem von Dosis und Applikationsart abhängigen Risiko zur Entwicklung einer inadäquaten Cortisolproduktion [152]. Kasuistische Berichte postulieren einen – letztendlich nicht sicher belegten – Zusammenhang zwischen einem Cortisolmangel und einer intraoperativen Hypotension bzw. einem Schockgeschehen [152, 153]. Die Steroiddauermedikation sollte daher präoperativ nicht pausiert und die übliche Steroiddosis auch am Morgen der Operation eingenommen werden ^**E69**^ (Tab. 31)

Ob Patientinnen und Patienten mit einer Steroiddauermedikation unterhalb der Cushing-Schwellendosis von einer zusätzlichen Steroid-Gabe profitieren, ist unklar [154]. Wegen der individuell sehr unterschiedlichen Reaktionen auf das operative Trauma und die unterschiedliche Suppression der endogenen Cortisolsynthese durch exogene Steroide kann auf der Basis von Expertenmeinung die einmalige Gabe von Steroiden erwogen werden ^**E70**^ (Tab. 31). Die Dauermedikation mit Steroiden sollte am OP-Tag fortgeführt werden. Bei der intraoperativen Gabe einer Stressdosis wird das Vorgehen basierend auf den Angaben in Tab. 32 empfohlen.

**Table 31:** 

	Empfehlung	Empfehlungsgrad	
E69	Eine Dauermedikation mit Kortikosteroiden SOLLTE fortgeführt werden.	B	⇑
E70	PatientInnen mit einer Kortikosteroid-Dauermedikation KÖNNEN perioperativ zusätzlich eine Cortisonsubstitution erhalten.	0	⇔

**Table 32:** 

Kleine operative Eingriffe	Mittlere operative Eingriffe	Große operative Eingriffe
25 mg Hydrocortison präoperativ	100 mg Hydrocortison/24 h	100 mg Hydrocortison/24 h 50 mg Hydrocortison/24 h (Tag 2) 25 mg Hydrocortison/24 h (Tag 3)

Die Kortikosteroidsubstitution bei hypophysären Eingriffen oder die Steroidgabe zur Therapie oder Prophylaxe des Hirnödems bei intrakraniellen Operationen müssen gesondert betrachtet werden.

### C.7 Analgetika und Co-Analgetika

Um das Risiko für eine perioperative Schmerzverstärkung zu verringern, sollten Analgetika und Co-Analgetika perioperativ nach Möglichkeit weitergegeben werden. Dies gilt insbesondere für gewöhnungsinduzierende Substanzen (z. B. Opioide und Gabapentinoide) zur Prophylaxe eines Entzugs. Der Umgang mit Nichtopioidanalgetika kann der gemeinsamen interdisziplinären Empfehlung „Perioperative Schmerztherapie mit Nichtopioidanalgetika“ entnommen werden [155]. Die Verwendung regionaler oder patientenkontrollierter Analgesieverfahren (PCA) soll berücksichtigt werden.

Bei Vormedikation mit dem Partialantagonisten Buprenorphin (Wirkung: agonistisch am μ‑Rezeptor, antagonistisch am κ‑Rezeptor) sollte die perioperative Gabe fortgeführt werden. Bei bereits vorbestehenden Behandlungen mit transdermalen Systemen (Fentanyl, Buprenorphin) ist eine individualisierte Therapieempfehlung zu berücksichtigen. In der Regel kann die analgetische Therapie mit transdermalen Systemen fortgeführt werden. Bei operativen Eingriffen, die die Resorption des Opioids aus dem subkutanen Fettgewebe stark beeinflussen (z. B. bei Eingriffen mit starken Volumenverschiebungen, Hyper- oder Hypoperfusion der Haut) sollten individualisierte Therapieüberlegungen erfolgen. Für eine Opioidrotation oder Umstellung auf einen alternativen Applikationsweg ist zu berücksichtigen, dass nach Entfernen des transdermalen Systems je nach Wirkstoff und Dosis für weitere 12 bis 24 h therapeutische Wirkspiegel zu erwarten sind.

Co-Analgetika wie Antidepressiva, Gabapentinoide oder orale Cannabinoide sollten perioperativ weitergegeben werden. Inhalative medizinische Cannabinoide sollten einer individualisierten Betrachtung unterzogen werden.

### C.8 Opioidabhängigkeit und Substanzen zur Behandlung von Substanzabhängigkeit

Zur Reduktion des Suchtdrucks, der sozialen Isolation und dem Vermeiden von Folgeschäden werden ca. 50 % der Heroinabhängigen substituiert. Die häufigsten Substanzen sind derzeit Levomethadon, Methadon, Buprenorphin und Morphin. Die Substitutionsbehandlung soll perioperativ fortgesetzt werden. Ausschließlich nach Rücksprache mit dem Substitutionsmediziner sollte bei verstärktem Suchtdruck die Substitutionsdosis angepasst werden.

Eine individualisierte Betrachtung erfordert die bereits präoperativ vorbestehende Behandlung von Patientinnen und Patienten mit Naltrexon, einem oralen Opioidantagonisten am µ‑ und κ‑Opioidrezeptor. Bei elektiven Eingriffen sollte präoperativ die Behandlung bereits mehrere Tage vor der Operation beendet werden. Eine enge Absprache mit der primär behandelnden Ärztin bzw. dem primär behandelnden Arzt sollte in Betracht gezogen werden.

## D. Schlussbemerkung

Die hier vorgelegten Konzepte zur präoperativen Evaluierung von erwachsenen Patientinnen und Patienten vor elektiven, nicht herz-thoraxchirurgischen Operationen stellen fachübergreifende Empfehlungen dar, die ein strukturiertes und gemeinsames Vorgehen ermöglichen und die Qualität der Behandlung von Patientinnen und Patienten verbessern sollen. Ihr Ziel ist es, durch transparente und verbindliche Absprachen eine hohe Patientinnen- und Patientenorientierung unter Vermeidung unnötiger Voruntersuchungen zu gewährleisten, präoperative Untersuchungsabläufe zu verkürzen sowie letztlich Kosten zu reduzieren. Dies bedeutet jedoch auch, dass für einzelne Patientinnen und Patienten individuelle Konzepte erstellt werden müssen.

Die vorliegenden gemeinsamen Empfehlungen von DGAI, DGCH sowie DGIM spiegeln den gegenwärtigen Kenntnisstand, aber auch die Meinungen von Experten wider, da nicht für jede Fragestellung wissenschaftliche Evidenz besteht. Daher wird eine regelmäßige Überprüfung und Aktualisierung der Empfehlungen erfolgen, sobald sich gesicherte neue Erkenntnisse ergeben.
